# CircItgb5 promotes synthetic phenotype of pulmonary artery smooth muscle cells via interacting with miR-96-5p and Uba1 in monocrotaline-induced pulmonary arterial hypertension

**DOI:** 10.1186/s12931-023-02480-9

**Published:** 2023-06-21

**Authors:** Hua Su, Huiqi Zhu, Sihao Wang, Yeping Li, Chao Yan, Jiaoyan Wang, Kejing Ying

**Affiliations:** Department of Pulmonary and Critical Care Medicine, Regional Medical Center for National Institute of Respiratory Diseases, School of Medicine, Sir Run Run Shaw Hospital, Zhejiang University, No. 3 Qingchun Road East, Hangzhou, China

**Keywords:** circItgb5, miR-96-5p, Uba1, Pulmonary arterial smooth muscle cells, Pulmonary arterial hypertension

## Abstract

**Background:**

Pulmonary arterial hypertension (PAH) is a rare but fatal cardiopulmonary disease mainly characterized by pulmonary vascular remodeling. Aberrant expression of circRNAs has been reported to play a crucial role in pulmonary vascular remodeling. The existing literature predominantly centers on studies that examined the sponge mechanism of circRNAs. However, the mechanism of circRNAs in regulating PAH-related protein remains largely unknown. This study aimed to investigate the effect of circItgb5 on pulmonary vascular remodeling and the underlying functional mechanism.

**Materials and methods:**

High-throughput circRNAs sequencing was used to detect circItgb5 expression in control and PDGF-BB-treated pulmonary arterial smooth muscle cells (PASMCs). Localization of circItgb5 in PASMCs was determined via the fluorescence in situ hybridization assay. Sanger sequencing was applied to analyze the circularization of Itgb5. The identification of proteins interacting with circItgb5 was achieved through a RNA pull-down assay. To assess the impact of circItgb5 on PASMCs proliferation, an EdU assay was employed. Additionally, the cell cycle of PASMCs was examined using a flow cytometry assay. Western blotting was used to detect biomarkers associated with the phenotypic switch of PASMCs. Furthermore, a monocrotaline (MCT)-induced PAH rat model was established to explore the effect of silencing circItgb5 on pulmonary vascular remodeling*.*

**Results:**

CircItgb5 was significantly upregulated in PDGF-BB-treated PASMCs and was predominately localized in the cytoplasm of PASMCs. In vivo experiments revealed that the knockdown of circItgb5 attenuated MCT-induced pulmonary vascular remodeling and right ventricular hypertrophy. In vitro experiments revealed that circItgb5 promoted the transition of PASMCs to synthetic phenotype. Mechanistically, circItgb5 sponged miR-96-5p to increase mTOR level and interacted with Uba1 protein to activate the Ube2n/Mdm2/ACE2 pathway.

**Conclusions:**

CircItgb5 promoted the transition of PASMCs to synthetic phenotype by interacting with miR-96-5p and Uba1 protein. Knockdown of circItgb5 mitigated pulmonary arterial pressure, pulmonary vascular remodeling and right ventricular hypertrophy. Overall, circItgb5 has the potential for application as a therapeutic target for PAH.

**Supplementary Information:**

The online version contains supplementary material available at 10.1186/s12931-023-02480-9.

## Introduction

Pulmonary hypertension (PH) is a fatal cardiopulmonary disease characterized by abnormally elevated pulmonary arterial pressure [1]. Pulmonary arterial hypertension (PAH) is a rare and complex disease that is classified as Group 1 PH by the World Health Organization [1]. The hallmark pathological change of PAH involves pulmonary vasoconstriction and pulmonary vascular remodeling (PVR), leading to right ventricular hypertrophy, right ventricular failure, and even death [2]. PVR is characterized by pulmonary artery medial thickening and extracellular matrix (ECM) deposition [3]. It is now understood that pulmonary arterial smooth muscle cells (PASMCs) constitute the primary cellular component of the medial layer of the pulmonary artery. The malignant proliferation of PASMCs predominantly accounts for PVR in PAH. Under pathological conditions, such as platelet derived growth factors beta polypeptide b (PDGF-BB) stimulation, PASMCs undergo a phenotypic switch from contractile to synthetic phenotype [4]. The synthetic phenotype of PASMCs is characterized by their abnormal proliferation and ECM production [5]. Although significant inroads have been achieved in better understanding the pathogenesis of PAH, its overall incidence and mortality rates remains high. Accordingly, uncovering new cellular processes and elucidating their mechanisms is essential to provide novel approaches for treating this disease.

Circular RNAs (circRNAs) have been widely identified in eukaryotic cells following the advent of deep sequencing technology [6]. It has been established that circRNAs regulate microRNAs (miRNAs) activity and protein expression via different mechanisms, adsorbing miRNAs by acting as sponges or protein scaffolds [7]. Lately, multiple studies have uncovered the sponging function of circRNAs in PH [2]. It has been shown that circSirtuin1 represses PASMCs proliferation, migration and autophagy to ameliorate pulmonary hypertension via targeting the miR-145-5p/protein kinase-B3 axis [8]. Hsa_circ_0002062 acts as a sponge for hsa-miR-942-5p and subsequently activates CDK6, further promoting PVR [9]. Moreover, our previous study elucidated that the circRNAs-miRNAs-mRNAs network played a vital role in PH progression [10]. However, there is a lack of comprehensive research on circRNA-interacted proteins, which limits our current understanding in this area.

Herein, we discovered a new circRNA named circItgb5, which was screened by high-throughput circRNAs sequencing. Whether circItgb5 is involved in the development of PAH remains unknown. We explored the role of circItgb5 in regulating monocrotaline (MCT)-induced rat PAH and its mechanism in regulating the phenotypic switch of PASMCs. Our results demonstrated that the expression of circItgb5 was significantly upregulated in PDGF-BB-treated PASMCs. Moreover, we identified a novel mechanism of action for circRNAs, whereby circItgb5 interacts with both miR-96-5p and Uba1 protein to regulate pulmonary vascular remodeling.

## Materials and methods

### Ethics

All animal experimental procedures were conducted in accordance with the principles approved by the Institutional Animal Care and Use Committee of Zhejiang University.

### Isolation and culture of PASMCs

Sprague–Dawley (SD) rats (SPF, male, 180–200 g, 4 weeks) were obtained from the Animal Experimental Center of Zhejiang University, China. Rats were kept in pathogen-free conditions with a 12 h light/dark cycle, at 25 °C and 60% relative humidity, with ad libitum access to standard food and drinking water. The enzymolysis method was used to isolate rat primary PASMCs as previously reported [11]. PASMCs were seeded in a 10 cm plate at 2 × 10^6^ cells/ml density in DMEM supplemented with 20% fetal bovine serum for 24 h. After 24 h, PASMCs were stimulated with recombinant rat PDGF-BB (PeproTech Inc, Rocky Hill, NJ) at 100 ng/ml for 12 h. After 12 h, PASMCs were washed in sterile PBS and collected for high-throughput circRNAs sequencing.

### High-throughput circRNAs sequencing

Total RNA from PASMCs in the control group (n = 3) and PDGF-BB group (n = 3) was isolated using RNA extract reagent (AP-MN-MS-RNA-50, Axygen, USA) according to the manufacturer's instructions. For circRNAs expression analysis, the reads were mapped to the genome using the STAR and DCC to identify the cirRNAs and to estimate the circRNAs expression. Differentially expressed circRNAs were identified using the edgeR software. R was used to generate the figures. CircRNAs showing altered expression with *P* < 0.05 and more than 1.5 fold change were considered differentially expressed circRNAs. The high-throughput circRNAs sequencing and circRNAs array analysis was performed by Geneseed Biotech (Guangzhou, China).

### RNA isolation and reverse transcription-quantitative polymerase chain reaction (RT-qPCR)

Total RNA was extracted from control and treated PASMCs using RNA extract reagent (AP-MN-MS-RNA-50, Axygen, USA). Genomic DNA (gDNA) was extracted using the TIANamp Genomic DNA Kit (Tiangen Biotech, China). The purity and concentration of RNA and DNA were quantified by spectrophotometry using a NanoDrop 2000 (Thermo Fisher Scientific). For the reverse transcription of RNA, 1 µg of the RNA sample was utilized, and the PrimeScript RT Reagent kit from Takara Biotechnology Co., Ltd. was employed. The reaction mixture was prepared to achieve a final volume of 20 µl. The reverse transcription process involved incubation at 16 °C for 30 min, followed by 42 °C for 30 min, and a final denaturation step at 85 °C for 5 min. For the RNase R treatment, 1 µg of RNA was incubated with 4 U of RNase R (R0301, Geneseed Biotech) at 37 °C for a duration of 30 min. After the treatment, the RNase R-treated RNA (1 µg) was subjected to reverse transcription using random primers. PCR was performed using a PCR Master Mix kit (cwbiotech, China). PCR products were used for Sanger sequencing. qPCR analyses were performed with the SYBR Premix Ex Taq™ II (RR820A, Takara, Japan) using the LightCycler480 Real-Time PCR system (Applied Biosystems, Foster City, CA). The qPCR protocol consisted of three steps: heating at 95 °C for 30 s, 40 cycles of denaturation at 95 °C for 5 s, and extension at 60 °C for 30 s. Finally, a melting curve analysis was performed with temperature settings of 95 °C for 5 s and 60 °C for 1 min. The reaction volume was 20 μl containing 1.6 μl of forward or reverse primers (10 μM), 2 μl of complementary DNA (cDNA) or gDNA templates, 6.4 μl of ddH_2_O, and 10 μl of SYBR. The CT value of target genes was normalized to 5sRNA or GAPDH using the 2^−ΔΔCT^ method. The primer sequences used in the study can be found in Additional file 1: Tables S1–S3.

### Western blotting

Protein was extracted and quantified using RIPA buffer (P0013B, Beyotime, China). Then the protein mixture was centrifuged at 12,000 × rpm for 20 min at 4 °C. BCA protein assay kit (Beyotime, China) was used to detect protein concentration. Protein (50 μg) was subjected to 12% SDS-PAGE and transferred to PVDF membrane (Millipore, USA). Membranes were incubated with the primary antibodies for 18 h at 4 °C. Primary antibodies included COL1A1 (1:1000, A16891, ABclonal, USA), SM22 (1:1000, A6760, ABclonal, USA), AGO2 (1:1000, C34C6, CST, USA), mTOR (1:1000, 7C10, CST, USA), ACE2 (1:1500, ab108252, Abcam, USA), Mdm2 (1:1000, ab259265, Abcam, USA), Ube2n (1:1000, ab25885, Abcam, USA), Uba1 (1:2000, ab264179, Abcam, USA), and GAPDH (1:4000, ab8245, Abcam, USA). HRP-conjugated secondary antibodies were incubated with the membranes for 2 h at room temperature. ECL was used to detect the immunoreactive bands, and densitometry of the blots was analyzed by ImageJ software.

### SiRNA, plasmid construction and cell transfection

CircItgb5 lentiviral small hairpin RNA (Lsh-circItgb5), lentiviral small hairpin RNA negative control (Lsh-NC), miR-96-5p mimic, miR-96-5p inhibitor, a mimic control, and an inhibitor control (iCon) were synthesized by RiboBio (Guangdong, China). Binding sites of miR-96-5p with circItgb5 or mTOR 3'UTR were predicted by Targetscan. pGL3-circItgb5 (wild-type, circItgb5-WT), pGL3-circItgb5-MUT1 (binding sequences mutated, circItgb5-MUT1), pGL3-circItgb5-MUT2 (binding sequences mutated, circItgb5-MUT2), pGL3-mTOR 3ʹUTR (wild-type, mTOR 3ʹUTR-WT), pGL3-mTOR 3ʹUTR MUT (binding sequences mutated, mTOR 3'UTR-MUT), pcDNA3.1(−)-Uba1 (pUba1), and pcDNA3.1(−) (Vector) were synthesized by Genechem (Shanghai, China). The luciferase reporter vectors were constructed by cloning the wild-type binding site sequence (or mutant) of circItgb5 or mTOR 3ʹUTR into the pGL3 plasmid. For siRNA transfection, 50 nM Lsh-NC or 50 nM Lsh-circItgb5 were mixed with 125 μl of OPTI-MEM by gentle pipetting. PASMCs were pretreated with 100 ng/ml PDGF-BB for 12 h. For the miR-96-5p inhibitor rescue experiment, 50 nM Lsh-NC or Lsh-circItgb5 was cotransfected with 50 nM iCon or miR-96-5p inhibitor for corresponding well. For the pUba1 rescue experiment, 1000 ng of vector or pUba1 was cotransfected with 50 nM Lsh-NC or Lsh-circItgb5 for corresponding well. The transfection mixture was incubated for 20 min and then directly added to the cells. The transfection reagent was removed after 6 h, and the cells were incubated for 24 h and then used as required. Sequences used in this part can be found in Additional file 1: Table S3.

### Luciferase assay

HEK-293 T cells were seeded in a 24-well plate at a density of 1 × 10^4^ cells/ml. After 24 h, HEK-293 T cells were cotransfected with 200 ng of target plasmids and 20 ng of Renilla plasmid along with 40 nM miCon or 40 nM miR-96-5p mimic using 1.5 μl of lipofectamine 3000 for each well. The luciferase activity was examined with a dual-luciferase assay kit (E2920, Promega, USA). The fluorescence intensity was measured with a GloMax 20/20 fluorescence detector (Shaanxi Sino-American Biotechnology Co., Shanxi, China).

### RNA immunoprecipitation (RIP) assay

The EZ-Magna RIP kit (17-701, Millipore, USA) was used for the RIP assay, following the manufacturer's instructions. PASMCs were cross-linked by treating with formaldehyde at 37 °C for 10 min. After washing with cold PBS, PASMCs were incubated in 4 ml cell lysis buffer for 5 min on ice. A 1.5 g aliquot of anti-AGO2 (2897, CST, USA) or anti-Uba1 antibody (ab264179, Abcam, USA) or anti-IgG (PP64B, Millipore) was conjugated to protein A/G magnetic beads overnight at 4^◦^C. A 100 ng aliquot of total RNA was then incubated with the antibody in an IP buffer supplemented with RNase and protease inhibitors. Then, PASMCs were incubated with RIP buffer containing conjugated-magnetic beads at 4 °C for 6 h, following DNase treatment for 30 min at room temperature. Beads were washed using the washing buffer, and protein was removed from the compounds via incubation with 0.1% SDS/0.5 mg/ml Proteinase K (30 min, 55 °C). RNA complexes were isolated using phenol–chloroform extraction (P1025, Solarbio) and analyzed via RT-PCR or RT-qPCR.

### Pull-down assay and mass spectrometry identification

RNA pull-down assay was used to identify the putative proteins that interacted with circItgb5 using a magnetic RNA‑protein pull‑down kit (20164, Thermo Fisher Scientific, USA). CHO cells were cultured in DMEM supplemented with 10% FBS. CircItgb5 and circItgb5-MS2 were constructed into pLC5-ciR plasmid. Then 5 µg CHO-circItgb5-MS2 plasmid or CHO-MS2-CP (control plasmid) were transfected in CHO cells using lipofectamine 3000 (Life Technologies). It is well-established that MS2-CP specifically binds to circItgb5 when tagged with MS2 to form the MS2-CP-circItgb5-MS2 complex. After 48 h, cells were washed with PBS three times. Then the cells were lysed in 800 µl lysis buffer containing 20 mM Tris–HCl at pH 7.5, 100 mM KCl, 5 mM MgCl2, 0.5% NP-40, protease inhibitors (Thermo Fisher Scientific, USA), RNase inhibitor (TaKaRa, Japan), and 10 mM DTT for 10 min on ice. The cell lysates were centrifuged at 10,000*g* for 15 min at 4 °C. The collected supernatant was then incubated with 100 μL protein A + G beads (B23201, bimake, USA). 1 ml of binding buffer was added, and the mixture was washed 3 times. Subsequently, 1 ml of the reaction solution and 5 μg of the appropriate antibody were added to the mixture at 4 °C and rotated for 2 h. Following the completion of the reaction, the sediment was washed with 1 ml binding buffer three times. Complexes bound with magnetic beads were separated by SDS-PAGE. The entire gel was stained with silver kit (P0017S, Beyotime, China) and then cut into pieces at the target sites. The cut gel was destined, reduced, and alkylated, followed by trypsin digestion. The mass spectrometry identification was conducted by Shanghai iProteome Biotechnology Co., Ltd (Shanghai, China).

### RNA fluorescence in situ hybridization (FISH)

Cy3-labeled α-SMA probe and Cy5-labeled circItgb5 probe were designed by Haokebio (Hangzhou, China). The signals from the labeled probes were detected using a FISH Kit (GeneChem, Shanghai, China). PASMCs were fixed in 4% paraformaldehyde, then incubated with a Cy3-labeled α-SMA probe and a Cy5-labeled circItgb5 probe overnight at 4 °C. DAPI was used to label the nuclei. Images were analyzed with a fluorescence microscope.

### Cell proliferation

The EdU assay was carried out with the EdU detection kit (RiboBio, Guangzhou, China). 2 × 10^3^ PASMCs were seeded into 96-well plates in the logarithmic growth phase. Each well was incubated with 50 µM EdU solution for 2 h and washed with PBS twice for 5 min. 4% paraformaldehyde was used to fix PASMCs at room temperature for 30 min, and the cells were incubated in 1 × Apollo staining solution for 30 min at room temperature. Finally, 1 × DAPI solution was added to each well and incubated at room temperature in the dark for 30 min. Fluorescence was detected using a fluorescence microscope (Olympus Corporation, Japan).

### Cell migration assay

24-Well transwell chambers (3422, Corning, Toledo, USA) were used for transwell assay. After 24 h of transfection, PASMCs were seeded into the upper chamber of 24-well plates at a density of 2 × 10^4^ with a serum-free medium. The bottom chambers were filled with a medium with 10% FBS. After 48 h of incubation, PASMCs on the underside were fixed with 1% formaldehyde solution and stained with crystal violet. Ten random fields of cells were counted.

### Flow cytometry assay

PASMCs were transfected with Lsh-NC, Lsh-circItgb5, iCon, miR-96-5p inhibitor, vector and pUba1. Then PASMCs were collected using trypsin and washed with PBS. Cell cycle was detected using propidium iodide kit (Beyotime Biotechnology, Shanghai, China). The cell cycle was detected with a flow cytometer (Bio-Rad, USA).

### Animal treatment

24 SD rats were assigned to four groups using simple randomization. SD rats in the control group were subcutaneously injected with the hank's balanced salt solution (HBSS) at 0.1 ml/kg dose. SD rats in the MCT group were subcutaneously injected with 60 mg/kg MCT (Sigma Chemicals, St. Louis, MO, USA). SD rats in the (MCT + Lsh-circItgb5) group were injected 3 × 10^8^ TU/mL Lsh-circItgb5 via the tail vein to knock down circItgb5 10 days before MCT injection. SD rats in the (MCT + Lsh-NC) group were injected 3 × 10^8^ TU/mL Lsh-NC via the tail vein 10 days before MCT injection. The rats were anesthetized with isoflurane and killed after 3 weeks. Hypoxic pulmonary hypertension (HPH) rat models were established as we previously reported [10]. Euthanasia was performed by intraperitoneal injection of 100 mg/kg sodium pentobarbital. Then, hemodynamic experiments were conducted. The method used to kill rats was based on the literature [10]. Part of the lung tissue was divided into two parts. The left lung tissue was stored in 4% paraformaldehyde for subsequent pathological examination, and the remaining tissue was immediately frozen in liquid nitrogen for subsequent RT-qPCR or western blotting. Heart tissues from three rats of each group were stored in 4% paraformaldehyde for HE staining, and the other three were separated for right ventricle remodeling assessment.

### Hemodynamic evaluation

Right ventricular systolic pressure (RVSP) was measured after corresponding treatment in rats. After the external right jugular vein was exposed, a 1.2 French pressure catheter (Scisense, Inc) was inserted and carefully advanced into the superior vena cava and the right ventricular vein. RVSP was continuously recorded for one minute. After the measurement of RVSP, the thorax was opened, and the heart was dissected and weighed to calculate the right ventricular hypertrophy index (RVHI). RVHI stands for the ratio of the right ventricular (RV) wall weight to the left ventricular (LV) wall plus septum weight (S) (RV/(LV + S)).

### Histopathological observation

Analysis of pulmonary artery morphology was conducted via Hematoxylin and Eosin (HE) staining, Immunohistochemistry (IHC) staining, and Masson staining. The right ventricular hypertrophy morphology was analyzed via HE staining and Masson staining. After fixation with 4% paraformaldehyde, the tissue was dehydrated, infiltrated, waxed, and embedded in paraffin. The sections were then subjected to HE and Masson staining. Masson staining was used to test collagen deposition in the pulmonary artery and heart. Paraffin-embedded lung sections were stained with α-SMA (1:100, ab124964, Abcam, USA). The ratio of small pulmonary arteries thickness and muscularization was calculated as previously described [12].

### Bioinformatics analyses

MiRNAs binding sites on circItgb5 were predicted by RNAhybrid (http://bibiserv.techfak.uni-bielefeld.de/rnahybrid/) and miRanda (http://www.microrna.org/microrna/home.do). Targets of miR-96-5p were predicted by TargetScan (https://www.targetscan.org). The circItgb5-miRNAs and miR-96-5p-mRNAs interaction networks were constructed by Cytoscape. The protein–protein interaction (PPI) network was constructed by STRING (https://cn.string-db.org).

### Statistical analysis

All data were presented as mean ± standard deviation (SD) obtained using SPSS 19.0 (Chicago, IL, USA) software. At least three biological replicates were performed for all experiments. A two-tailed Student's t-test was used to compare the differences between two groups, whereas the comparison among multiple groups was conducted using one-way ANOVA with a Tukey's post hoc test. A *P-*value < 0.05 was statistically significant.

## Results

### CircItgb5 is significantly upregulated in PDGF-BB-treated PASMCs

**Fig. 1:**
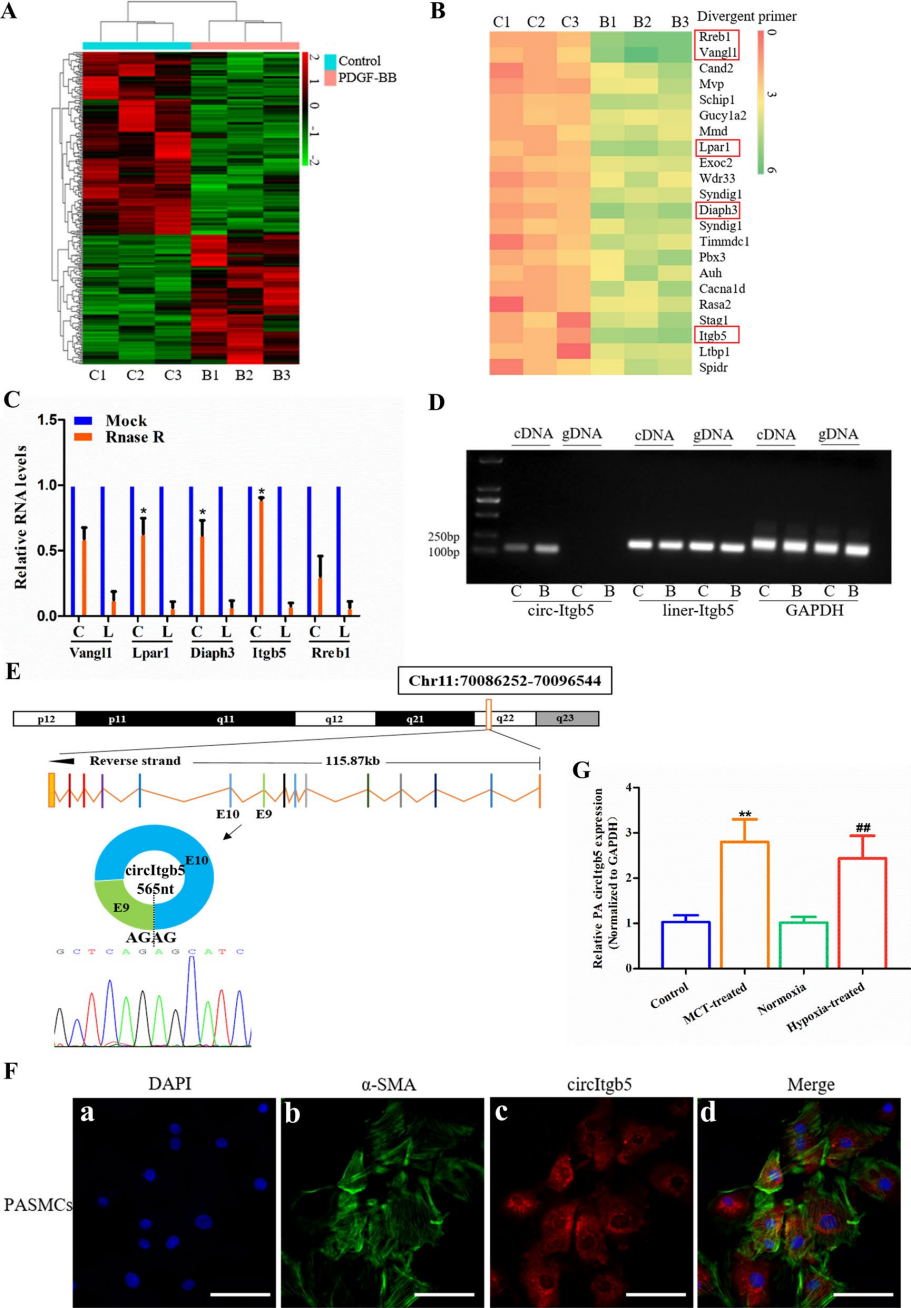
CircItgb5 is upregulated by PDGF-BB stimulation in PASMCs. **A** Heatmap showing a subset of circRNAs differentially expressed in PASMCs treated with PDGF-BB (100 ng/ml) for 12 h using high-throughput circRNAs sequencing. C, control; B, PDGF-BB. Fold change > 2, FKPM > 0.5 and FDR < 0.05. The colors in the heatmap represent fold change. **B** Validation of top 22 upregulated circRNAs from circRNAs-seq. RT-qPCR was conducted following PDGF-BB treatment for 12 h. C, control; B, PDGF-BB. Fold change > 2, FDR < 0.05. The top five upregulated circRNAs were highlighted with red frame. The colors in the heatmap represent fold change. **C** Expression of the top five upregulated circRNAs and the corresponding linear RNAs were detected by RT-qPCR following RNase R treatment. C, circular; L, linear. *0.01 ≤ *P* ≤ 0.05 versus Mock. **D** The presence of circItgb5 was detected by RT-PCR in PASMCs. Divergent primers amplified circItgb5 from cDNA, but not from gDNA. GAPDH was used as a positive control. C, control; B, PDGF-BB; cDNA, complementary DNA; gDNA, genomic DNA. **E** Schematic illustration showing exon 9 to 10 of Itgb5 circularization to form circItgb5 (black arrow). The sequence of circItgb5 was analyzed by Sanger sequencing. **F** The FISH assay was conducted to detect circItgb5 in PASMCs using Cy3-labeled probes (circItgb5). Nuclei were stained with DAPI. α-SMA is a characteristic marker of PASMCs. DAPI, 2-(4-amidinophenyl)-6-indolecarbamidine dihydrochloride. Original magnification, × 400; Scale bar = 50 μm. **G** RT-qPCR was conducted to detect circItgb5 expression in PA of MCT-treated rats and hypoxia-treated rats. PA, pulmonary arteries. n = 6. **0.001 ≤ *P* ≤ 0.009 versus control, ^##^0.001 ≤ *P* ≤ 0.009 versus normoxia. All data are presented as means ± SD (n = 3 biological replicates)

PDGF-BB is a vital cytokine and has been reported to induce phenotypic switch in PASMCs, causing them to transition from contractile phenotype to synthetic phenotype, thus contributing to the progression of PAH [4]. To investigate the effect of PDGF-BB on circRNAs expression in PASMCs, two independently isolated batches of PASMCs were prepared for high-throughput circRNAs sequencing. A total of 4402 circRNAs were detected by quantitative analysis (Fig. 1A). Then, the top 22 upregulated circRNAs were selected for further validation, and 5 circRNAs (circReb1, circVangl1, circLpar1, circDiaph3, circItgb5) were significantly upregulated with foldchange > 2.5 (Fig. 1B). Further, circItgb5 was resistant to RNase R, while Itgb5 mRNA was significantly reduced after RNase R treatment (Fig. 1C). CDNA and gDNA were used as templates to detect circular and linear Itgb5. CircItgb5 was amplified using divergent primers in the cDNA template, while no amplification products were detected in gDNA (Fig. 1D). However, the abundance of circItgb5 in pulmonary artery endothelial cells (PAECs) was very low (see Additional file 2: Fig. S1). After obtaining the RT-PCR product of circItgb5, Sanger sequencing was performed, confirming the presence of a head-to-tail splicing structure in the sequence (Fig. 1E). As shown in Fig. 1F, circItgb5 was predominately localized in the cytoplasm of PASMCs. Moreover, circItgb5 expression was about 1.3-fold higher in pulmonary arteries in MCT-PAH rats than in HPH rats (Fig. 1G). CircItgb5 expression was also upregulated in MCT-induced lungs (see Additional file 2: Fig. S1).

### Knockdown of circItgb5 attenuates MCT-induced pulmonary vascular remodeling and right ventricular hypertrophy

**Fig. 2:**
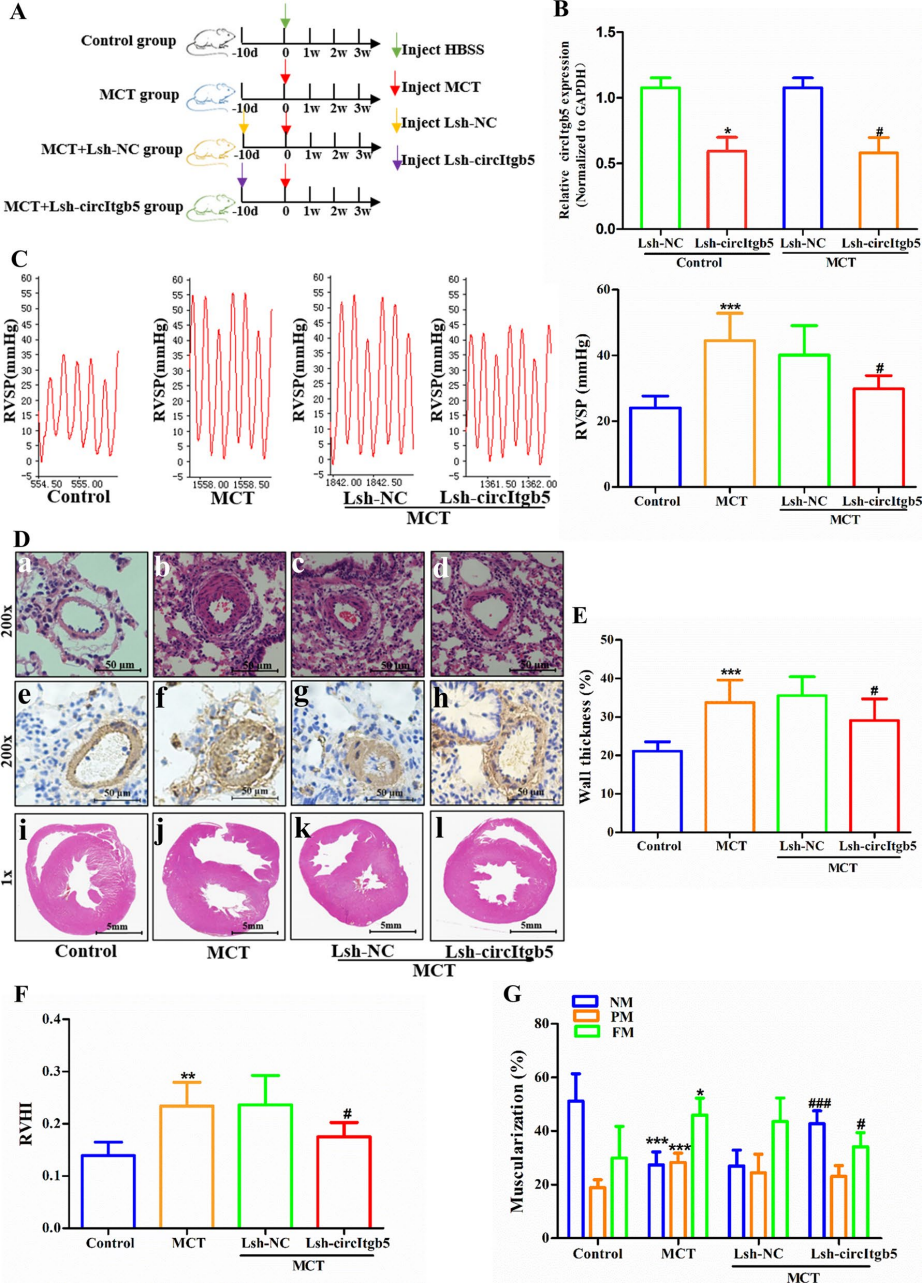
Knockdown of circItgb5 in vivo attenuates pulmonary hypertension, pulmonary artery remodeling and right ventricle hypertrophy. **A** SD rats were divided into four groups: control group, MCT group, (Lsh-NC + MCT) group, and (Lsh-circItgb5 + MCT) group. HBSS, hank's balanced salt solution. n = 6. **B** RT-qPCR was used to detect circItgb5 expression when it was knocked down in PA. n = 6. *0.01 ≤ *P* ≤ 0.05 versus (Lsh-NC + control), ^#^0.01 ≤ *P* ≤ 0.05 versus (Lsh-NC + MCT). **C** RVSP in the four groups was shown (n = 6). Scale bar = 1 s. ***P ≤ 0.001 versus control, ^#^0.01 ≤ *P* ≤ 0.05 versus (Lsh-NC + MCT). **D** Morphological analysis of small pulmonary arteries was performed using HE staining (**a**–**d**) and IHC staining (anti-α-SMA, **e**–**h**). Original magnification, × 200; scale bar = 50 µm. HE staining detected right ventricle hypertrophy (**i**–**l**). Original magnification, × 1; scale bar = 5 mm. **E** Wall thickness (%) of small pulmonary arteries. n = 6. ***P ≤ 0.001 versus control, ^#^0.01 ≤ *P* ≤ 0.05 versus (Lsh-NC + MCT). **F** Changes in the RVHI. RVHI = RV/(LV + S), RV, right ventricle; LV, left ventricle; S, ventricular septum. RVHI, right ventricular hypertrophy index. n = 6. **0.001 ≤ *P* ≤ 0.009 versus control, ^#^0.01 ≤ *P* ≤ 0.05 versus (Lsh-NC + MCT). **G** Muscularization of pulmonary small arteries. NM, nonmuscularized; PM, partially muscularized; FM, fully muscularized. n = 6. *0.01 ≤ *P* ≤ 0.05 versus control, ***P ≤ 0.001 versus control, ^#^0.01 ≤ *P* ≤ 0.05 versus (Lsh-NC + MCT), ^###^*P* ≤ 0.001 versus (Lsh-NC + MCT)

To further investigate the effect of circItgb5 in vivo, a MCT-induced PAH model was constructed and Lsh-circItgb5 was injected into rats to silence circItgb5 (Fig. 2A). Lsh-circItgb5 significantly downregulated circItgb5 expression in the pulmonary arteries of PAH rats (Fig. 2B). The mean RVSP was significantly higher in the MCT group than in the control group. Lsh-circItgb5 induced a decrease in the mean RVSP compared to the (Lsh-NC + MCT) group (29.83 ± 3.67 mmHg vs. 40.15 ± 8.15 mmHg, *P* < 0.05, Fig. 2C). Additionally, HE staining and immunostaining with α-SMA results indicated that knockdown of circItgb5 attenuated MCT-induced PVR and RVHI (Fig. 2D–F). In the control group, 51.15 ± 9.34% of the arterioles were nonmuscularized (NM) vessels, and 29.96 ± 10.79% were fully muscularized (FM) vessels. FM vessels occupied a greater proportion (43.60 ± 7.98%) in the (Lsh-NC + MCT) group, while NM vessels showed a lower proportion (26.95 ± 5.41%) (Fig. 2G). Compared to the (Lsh-NC + MCT) group, Lsh-circItgb5 treatment increased the proportion of NM vessels, and decreased the proportion of FM vessels in the (Lsh-circItgb5 + MCT) group (Fig. 2G). However, no inhibition effect was observed on the mean RVSP and vascular remodeling in the control group when circItgb5 was silenced (see Additional file 2: Fig. S2).

**Fig. 3:**
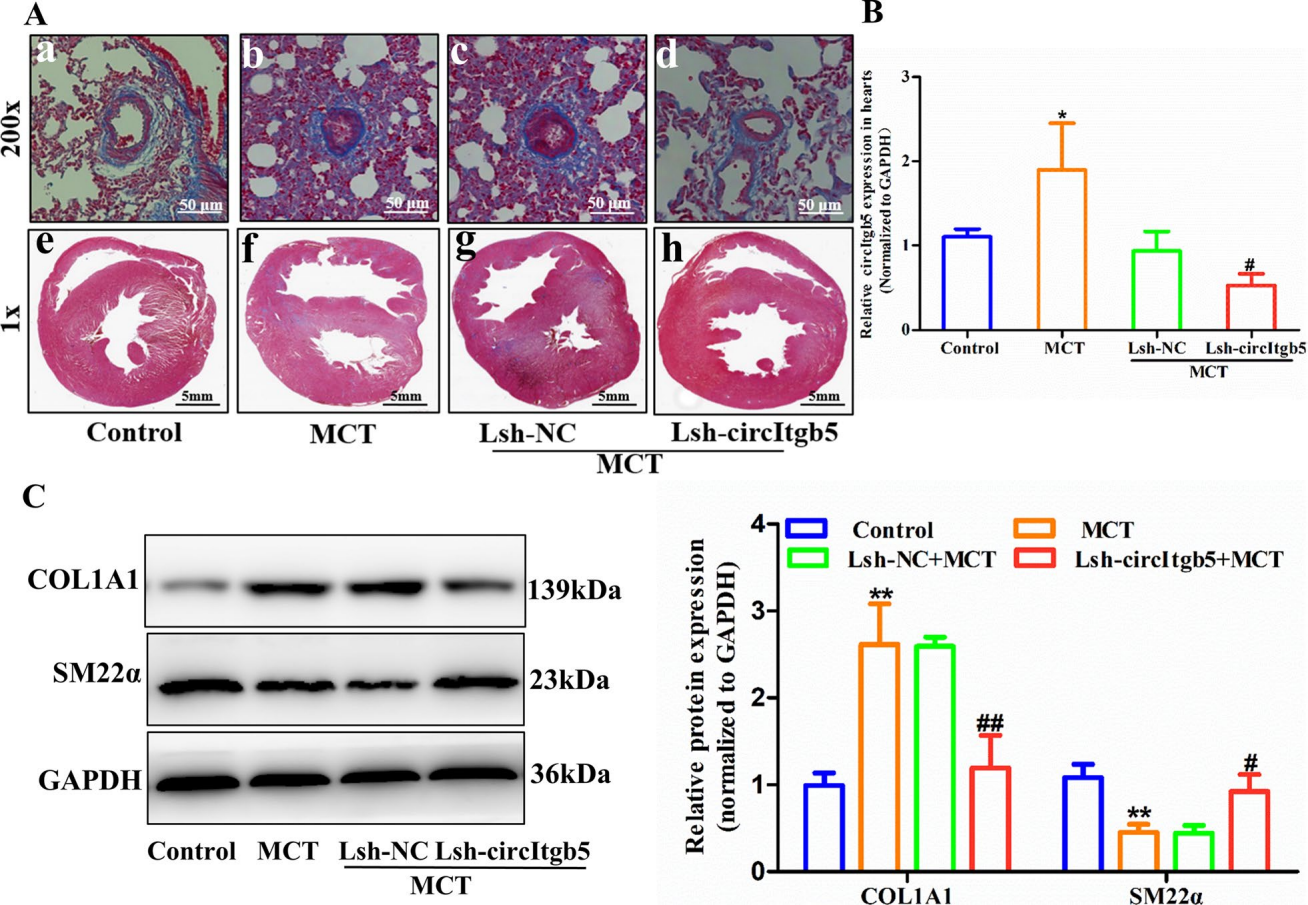
Silencing of circItgb5 in vivo decreases ECM deposition in small pulmonary arteries and the right ventricle. **A** Masson staining of small pulmonary arteries (original magnification, × 200; scale bar = 50 µm) and hearts (original magnification, × 1; scale bar = 5 mm) after knockdown of circItgb5 in vivo. **B** CircItgb5 expression in the heart of rats. n = 6. *0.01 ≤ *P* ≤ 0.05 versus control, ^#^0.01 ≤ *P* ≤ 0.05 versus (Lsh-NC + MCT). **C** Protein expression of COL1A1 and SM22α in pulmonary arteries was detected by western blotting. **0.001 ≤ *P* ≤ 0.009 versus control, ^#^0.01 ≤ *P* ≤ 0.05 versus (Lsh-NC + MCT), ^##^0.001 ≤ *P* ≤ 0.009 versus (Lsh-NC + MCT). The data are presented as means ± SD (n = 3 biological replicates)

Masson staining revealed that knockdown of circItgb5 mitigated the degree of fibrosis of pulmonary arteries and heart (Fig. 3A). The cardiac expression of circItgb5 was significantly increased with MCT treatment and knocked down by Lsh-circItgb5 (Fig. 3B). Compared to the (Lsh-NC + MCT) group, Lsh-circItgb5 restrained the expression of COL1A1 and augmented SM22α levels in pulmonary arteries (Fig. 3C).

### CircItgb5 promotes synthetic phenotype of PASMCs

**Fig. 4:**
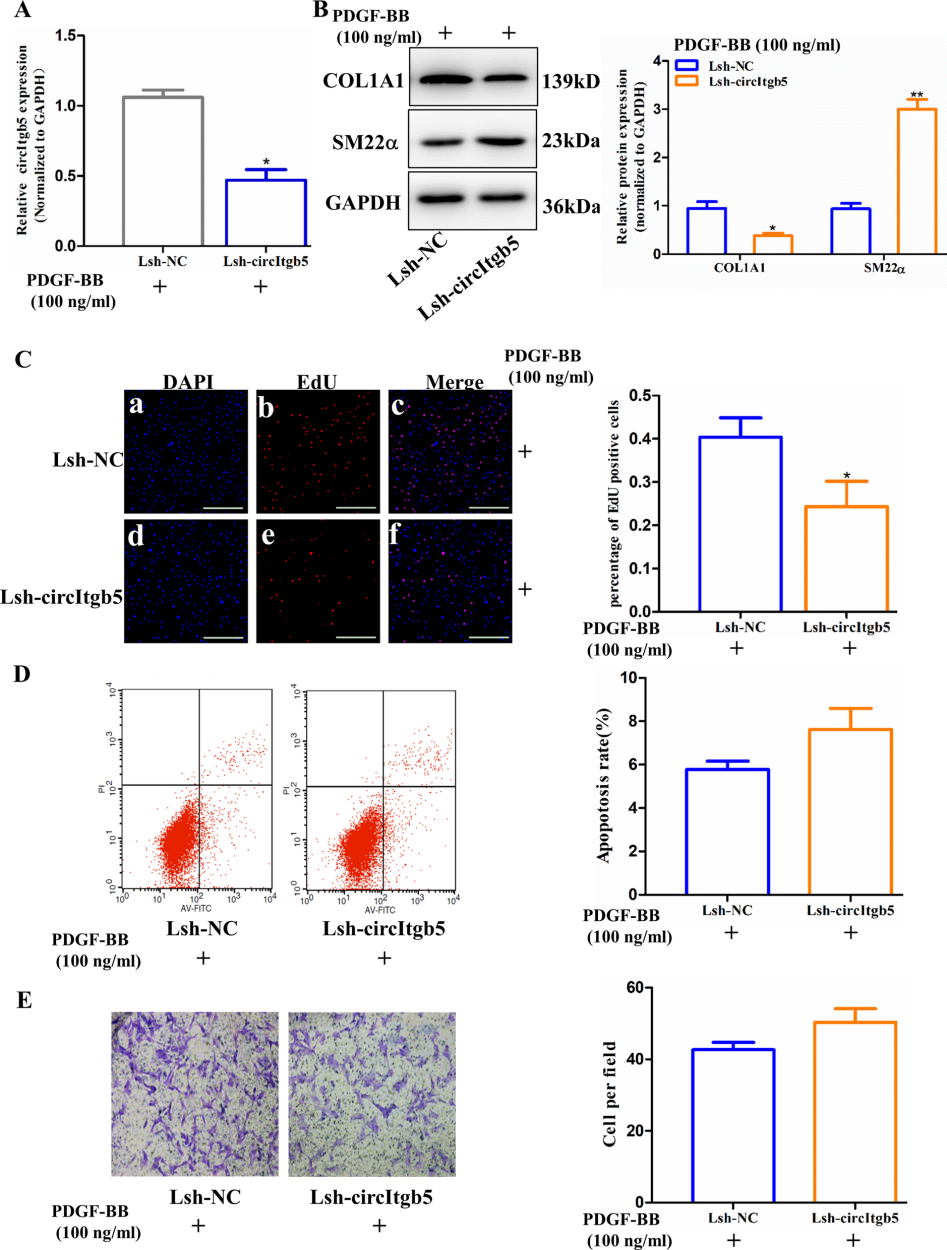
Silencing circItgb5 inhibits synthetic phenotype of PASMCs. **A** RT-qPCR was conducted to detect circItgb5 level when circItgb5 was inhibited by lentiviral shRNA in vitro. *0.01 ≤ *P* ≤ 0.05 vs Lsh-NC. **B** Expression of COL1A1 and SM22α was detected by western blotting. *0.01 ≤ *P* ≤ 0.05 versus Lsh-NC, **0.001 ≤ *P* ≤ 0.009 versus Lsh-NC. **C** PASMCs proliferation was detected using EdU assay. *0.01 ≤ *P* ≤ 0.05 versus Lsh-NC. Scale bar = 100 μm. **D** Flow cytometric analysis for quantification of early apoptotic cells. **E** Transwell assay was used to detect the migration ability of PASMCs. All data are presented as means ± SD (n = 3 biological replicates)

To explore the effect of circItgb5 on PASMCs, Lsh-circItgb5 was transfected into PASMCs. Lsh-circItgb5 significantly decreased circItgb5 expression in PASMCs (Fig. 4A). Silencing of circItgb5 induced PASMCs to transition to contractile phenotype (Fig. 4B). Moreover, the knockdown of circItgb5 declined the proportion of EdU-positive cells (Fig. 4C). However, inhibition of circItgb5 did not significantly affect the apoptosis rate and migration ability of PASMCs (Fig. 4D, E).

### CircItgb5 acts as a competing endogenous RNA to increase mTOR level by sequestering miR-96-5p

**Fig. 5:**
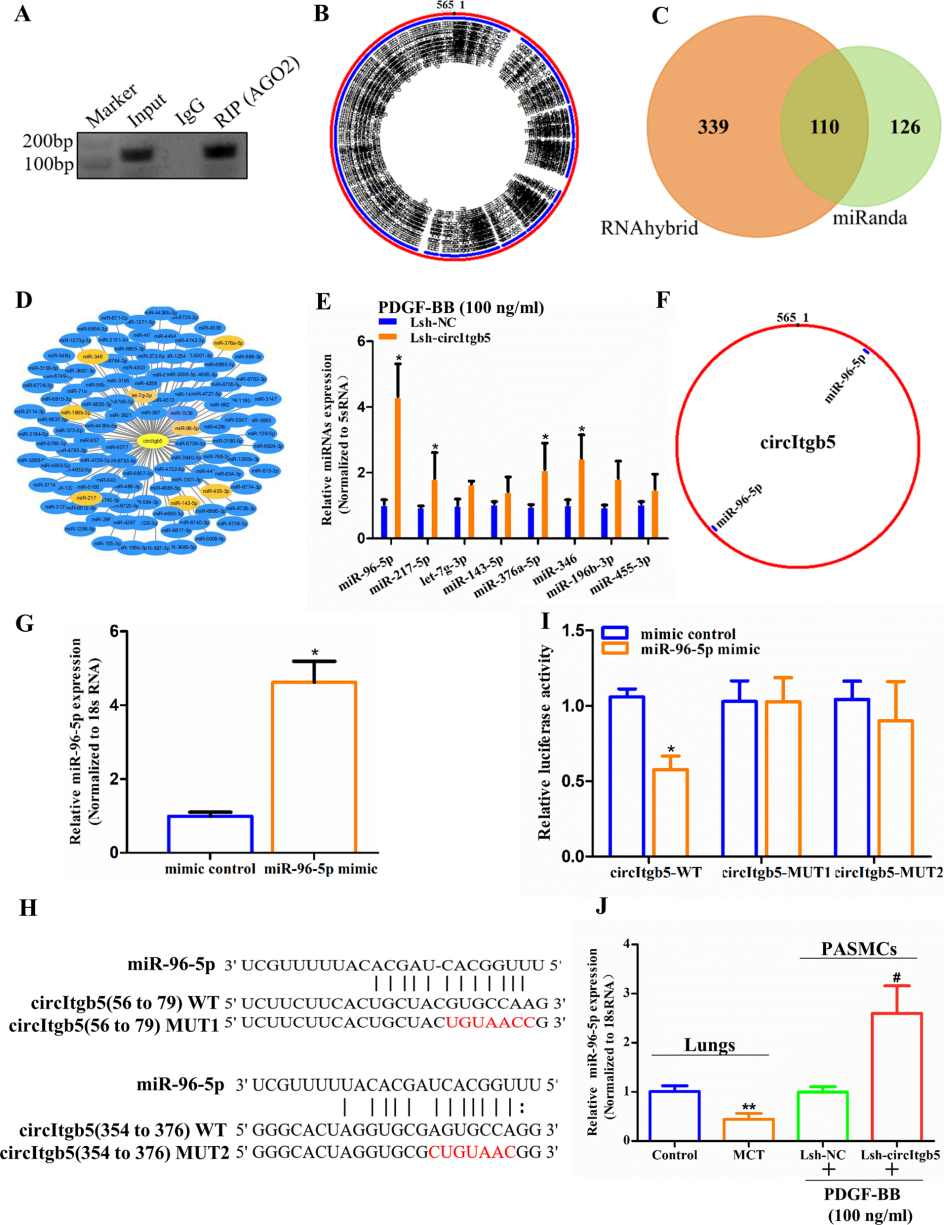
CircItgb5 serves as a sponge for miR-96-5p. **A** Total RNA was extracted from PASMCs and immunoprecipitated by AGO2 or IgG antibody. CircItgb5 in the immunoprecipitate was determined by RT-PCR using agarose gel electrophoresis. **B** Schematic illustration showed the binding of miRNAs on circItgb5 using CirMIR1.0. **C** MiRNAs interacted with circItgb5 were predicted by RNAhybrid and miRanda. **D** A cytoscape drawing circItgb5-miRNAs interaction network. Eight miRNAs were previously reported to be related to PH (shown in orange). **E** The selected eight miRNAs (from **D**) were validated by RT-qPCR. *0.01 ≤ *P* ≤ 0.05 versus Lsh-NC. **F** Schematic drawing showed the putative binding sites for miR-96-5p on circItgb5. **G** RT-qPCR was used to detect the relative level of miR-96-5p. *0.01 ≤ *P* ≤ 0.05 versus mimic control. **H**, **I** The luciferase activities of wild-type or mutant pGL3-circItgb5 in 293 T cells transfected with miR-96-5p mimic or mimic control. The red letter represents mutated sequences. The mutation principle: G to U, U to G, C to A, A to C. *0.01 ≤ *P* ≤ 0.05 versus mimic control. **J** Relative miR-96-5p level was detected in lungs of rats and PASMCs after the corresponding treatment. **0.001 ≤ *P* ≤ 0.009 versus control, ^#^0.01 ≤ *P* ≤ 0.05 versus Lsh-NC. All data are presented as means ± SD (n = 3 biological replicates)

It has been reported that circRNAs could serve as a miRNA sponge to regulate downstream target genes. Argonaute2 (AGO2) is the core protein of the RNA-induced silencing complexes that binds miRNAs to repress the expression of target mRNAs [13]. Therefore, we explored the interaction between circItgb5 and AGO2 using AGO2-RIP. The results displayed that circItgb5 could be immunoprecipitated by AGO2 (Fig. 5A). Then, CirMIR1.0 was used to predict miRNAs binding with circItgb5 (Fig. 5B). 110 candidate miRNAs were identified by overlapping the predicted miRNAs recognition elements in the circItgb5 sequence using miRanda and RNAhybrid (Fig. 5C). Among the candidate miRNAs, eight were previously reported to play a crucial role in PH (Fig. 5D) [5, 14]. In the present study, miR-96-5p was the most significantly upregulated miRNA when circItgb5 was knocked down (Fig. 5E). Two binding sites of miR-96-5p were predicted on circItgb5 (Fig. 5F). MiR-96-5p was significantly upregulated with miR-96-5p mimic treatment (Fig. 5G). To confirm whether the predicted binding motifs were indeed responsible for the interaction between miRNAs and circItgb5, luciferase reporter constructs of circItgb5-MUT1 plasmid and circItgb5-MUT2 plasmid were generated (Fig. 5H). A dual-luciferase reporter assay was performed to evaluate the effect of circItgb5 on miR-96-5p activity. The results showed that luciferase activity was reduced in 293 T cells co-transfected with miR-96-5p and WT-circItgb5 but not in 293 T cells transfected with circItgb5-MUT1 or circItgb5-MUT2 (Fig. 5I). As shown in Fig. 5J, miR-96-5p level was increased after the knockdown of circItgb5 in PASMCs and decreased in MCT-treated lungs.

**Fig. 6:**
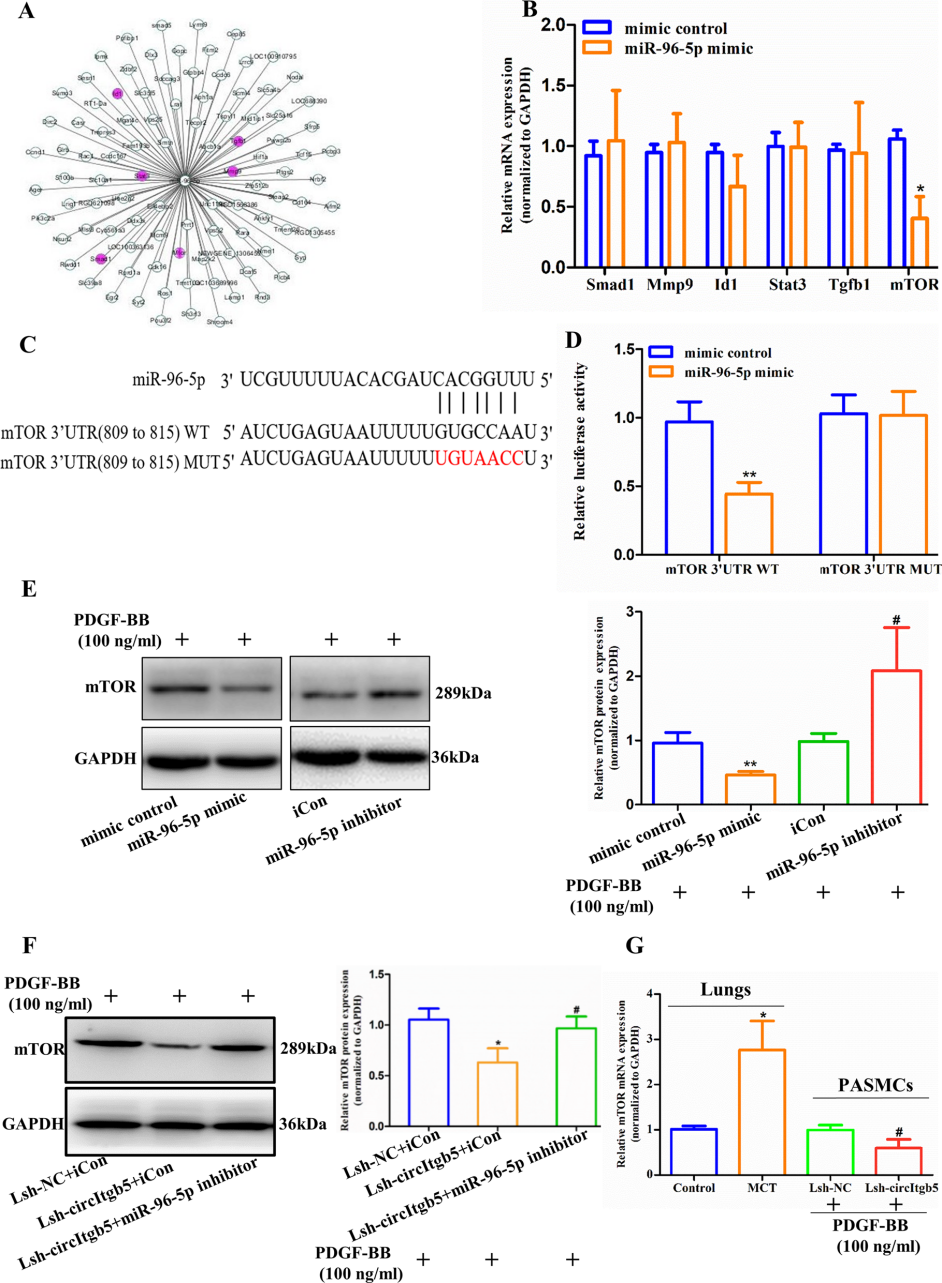
CircItgb5 regulates mTOR expression through sponging miR-96-5p. **A** A cytoscape was used to visualize miR-96-5p-target genes interaction based on TargetScan. Six genes highlighted in purple were previously reported to be related to PH. **B** Expression of the selected six genes in PASMCs was determined by RT-qPCR. *0.01 ≤ *P* ≤ 0.05 versus mimic control. **C**, **D** The luciferase activities of wild-type (WT) or mutant (MUT) pGL3-mTOR 3'UTR in 293 T cells cotransfected with miR-96-5p mimic or mimic control. The red letter represents mutated sequences. The mutation principle: G to U, U to G, C to A, A to C. **0.001 ≤ *P* ≤ 0.009 versus mimic control. **E** mTOR expression was determined by western blotting after the corresponding treatment in PASMCs. **0.001 ≤ *P* ≤ 0.009 versus mimic control, ^#^0.01 ≤ *P* ≤ 0.05 versus iCon. iCon, inhibitor control. **F** mTOR expression was determined by western blotting after the miR-96-5p inhibitor rescue experiment in PASMCs. *0.01 ≤ *P* ≤ 0.05 versus (Lsh-NC + iCon), ^#^0.01 ≤ *P* ≤ 0.05 versus (Lsh-circItgb5 + iCon). **G** Relative mTOR level was detected in the lungs and PASMCs after the corresponding treatment. *0.01 ≤ *P* ≤ 0.05 versus control, ^#^0.01 ≤ *P* ≤ 0.05 versus Lsh-NC. All data are presented as means ± SD (n = 3 biological replicates)

Further analysis was conducted to explore the target genes of miR-96-5p using TargetScan. As a result, six predicted target genes were identified, which have been associated with PH (Fig. 6A) [15, 16]. Therefore, we selected the six predicted target genes for further validation. Among them, mTOR mRNA was significantly decreased when miR-96-5p was overexpressed (Fig. 6B). Bioinformatics analyses indicated that the binding sites of mTOR 3'UTR on miR-96-5p overlapped with those of circItgb5 (Fig. 6C). A dual-luciferase reporter assay was performed to evaluate the effect of mTOR 3'UTR on miR-96-5p activity. The results demonstrated that luciferase activity was reduced when co-transfected with miR-96-5p and mTOR 3'UTR-WT but not when transfected with mTOR 3'UTR-MUT (Fig. 6D). Expression of mTOR was decreased when miR-96-5p was overexpressed, whereas its expression was increased when miR-96-5p was inhibited (Fig. 6E). Moreover, the knockdown of circItgb5 decreased the mTOR level, while this effect was partially rescued by the miR-96-5p inhibitor (Fig. 6F). Overall, these results indicated that circItgb5 could competitively sponge miR-96-5p with mTOR mRNA. The expression of mTOR mRNA was decreased after the knockdown of circItgb5 and increased by MCT-treatment (Fig. 6G).

### CircItgb5 interacts with Uba1

**Fig. 7:**
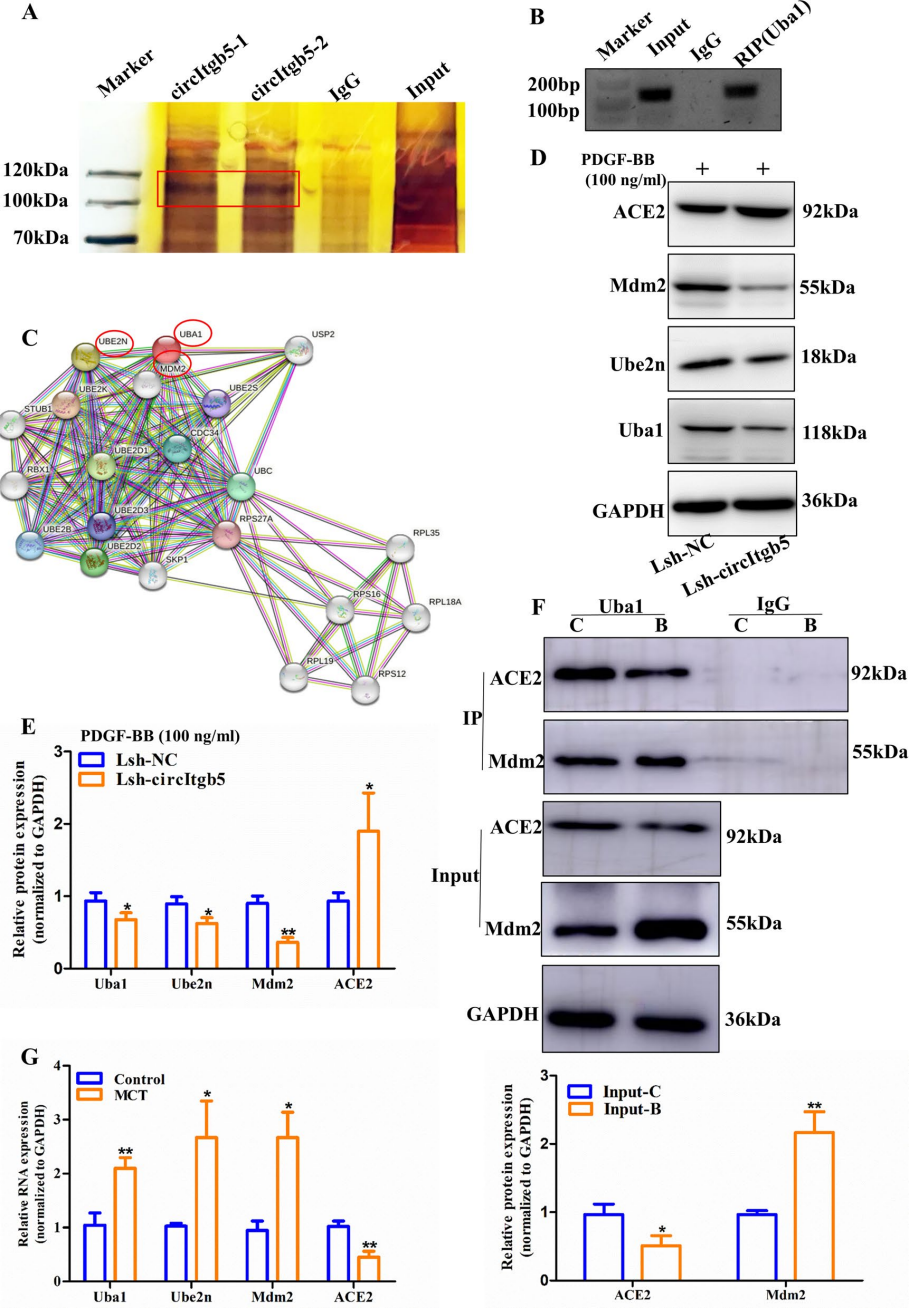
CircItgb5 interacts with the Uba1 protein. **A** CHO cells transfected with circItgb5-MS2 plasmid can specifically pull-down Uba1 protein using silver staining. Strips of Uba1 protein were highlighted with red frame. **B** CircItgb5 RT-PCR products were determined by agarose gel electrophoresis using anti-Uba1 antibody. **C** The PPI network was used to analyze proteins that interacted with Uba1; the red circle represents Ube2n (E2) and Mdm2 (E3). PPI, protein–protein interaction; E, enzyme. **D**, **E** Protein level of ACE2, Mdm2, Ube2n, and Uba1 was detected in PASMCs. *0.01 ≤ *P* ≤ 0.05 versus Lsh-NC, **0.001 ≤ *P* ≤ 0.009 versus Lsh-NC. **F** PASMCs extracts were immunoprecipitated with anti-Uba1 or IgG and immunoblotted with anti-ACE2 and anti-Mdm2. Input protein was immunoblotted with antibodies against ACE2 and Mdm2. C, control; B, PDGF-BB. *0.01 ≤ *P* ≤ 0.05 versus Input-C, **0.001 ≤ *P* ≤ 0.009 versus Input-C. **G** mRNA expression of Uba1, Ube2n, Mdm2, and ACE2 in the lungs. *0.01 ≤ *P* ≤ 0.05 versus control, **0.001 ≤ *P* ≤ 0.009 versus control. All data are presented as means ± SD (n = 3 biological replicates)

To identify potential proteins interacting with circItgb5, we performed circItgb5 RNA pull-down assay and mass spectrometry identification. We observed specific circItgb5 pull-down protein with the enrichment of Uba1 (Fig. 7A). CircItgb5 was also immunoprecipitated by Uba1 (Fig. 7B). To explore the proteins interacting with Uba1, we constructed a PPI network. The results demonstrated that Uba1 interacted with Ube2n and Mdm2 (Fig. 7C). It is well-established that Ube2n and Mdm2 both participated in ubiquitin signaling. A previous study reported that Mdm2 ubiquitinated ACE2, thus leading to ACE2 degradation [17]. To explore whether circItgb5 could influence ACE2 expression, we knocked down circItgb5 to detect its expression. Interestingly, knockdown of circItgb5 inhibited expression of Uba1, Ube2n and Mdm2, but promoted ACE2 expression (Fig. 7D, E). We further confirmed that Mdm2 and ACE2 were immunoprecipitated by Uba1 in PASMCs. Mdm2 level was increased, while ACE2 expression was decreased via PDGF-BB stimulation (Fig. 7F). The expression of Uba1, Ube2n and Mdm2 was increased while the ACE2 level was decreased following MCT-treatment (Fig. 7G).

### CircItgb5 promotes synthetic phenotype of PASMCs through interacting with miR-96-5p and Uba1

**Fig. 8:**
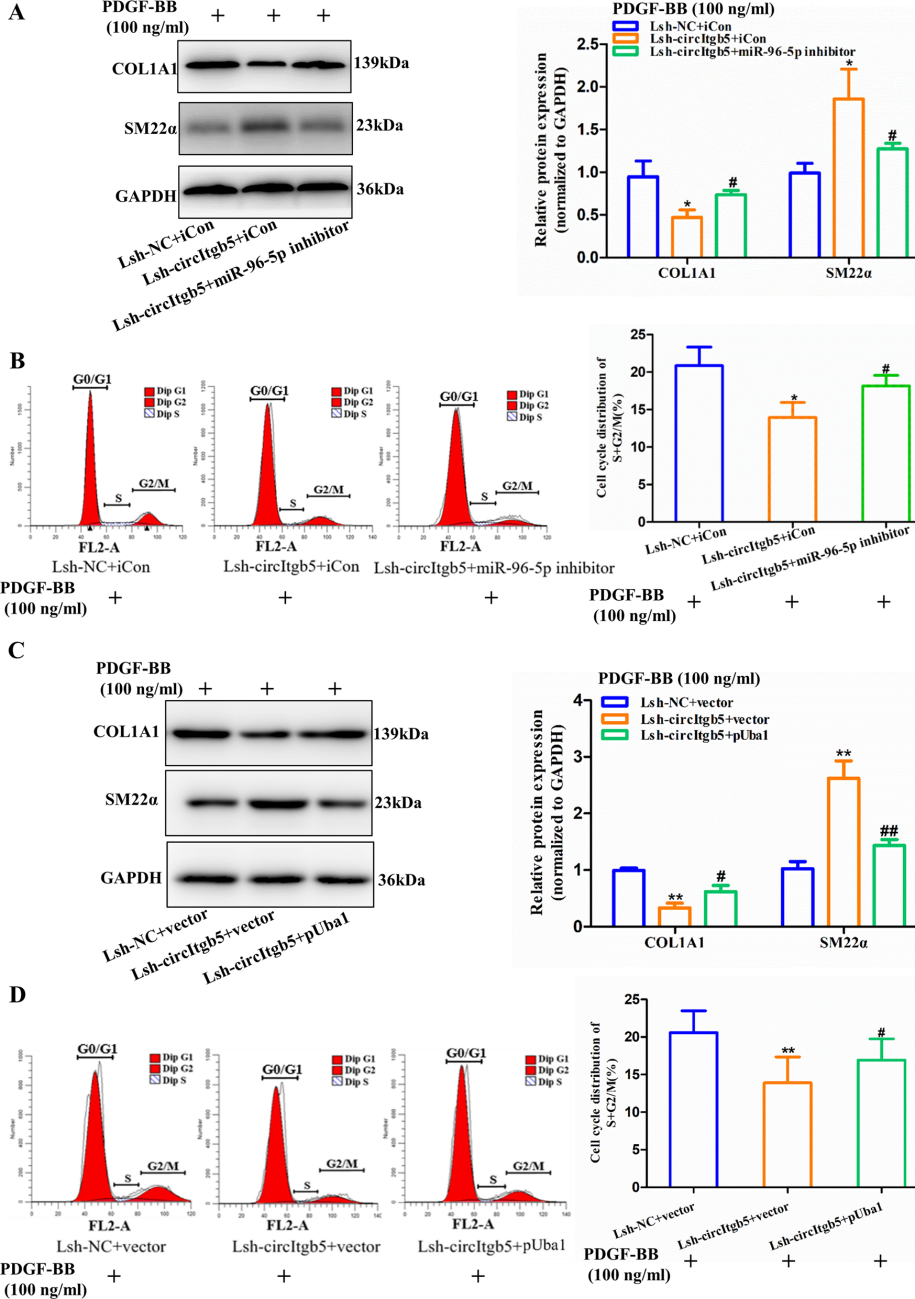
CircItgb5 promotes synthetic phenotype of PASMCs by interacting with miR-96-5p and Uba1. **A**, **C** Protein expression of COL1A1 and SM22α was determined after the corresponding treatment. *0.01 ≤ *P* ≤ 0.05 versus (Lsh-NC + iCon), **0.001 ≤ *P* ≤ 0.009 versus (Lsh-NC + vector), ^#^0.01 ≤ *P* ≤ 0.05 versus (Lsh-circItgb5 + iCon) or (Lsh-circItgb5 + vector), ^##^0.001 ≤ *P* ≤ 0.009 versus (Lsh-circItgb5 + vector). **B**, **D** The cell cycle of PASMCs was measured by flow cytometry. *0.01 ≤ *P* ≤ 0.05 versus (Lsh-NC + iCon), **0.001 ≤ *P* ≤ 0.009 versus (Lsh-NC + vector), ^#^0.01 ≤ *P* ≤ 0.05 versus (Lsh-circItgb5 + iCon) or (Lsh-circItgb5 + vector). All data are presented as means ± SD (n = 3 biological replicates)

Rescue experiments were conducted to confirm the effect of circItgb5 on PASMCs. Lsh-circItgb5 significantly inhibited COL1A1 expression but increased SM22α level, while miR-96-5p inhibitor and pUba1 partially reversed this effect (Fig. 8A, C). Similarly, Lsh-circItgb5 significantly inhibited the proliferation of PASMCs, while miR-96-5p inhibitor and pUba1 partially reversed this effect (Fig. 8B, D). The above results indicated that circItgb5 promoted synthetic phenotype of PASMCs by interacting with miR-96-5p and Uba1.

## Discussion

**Fig. 9:**
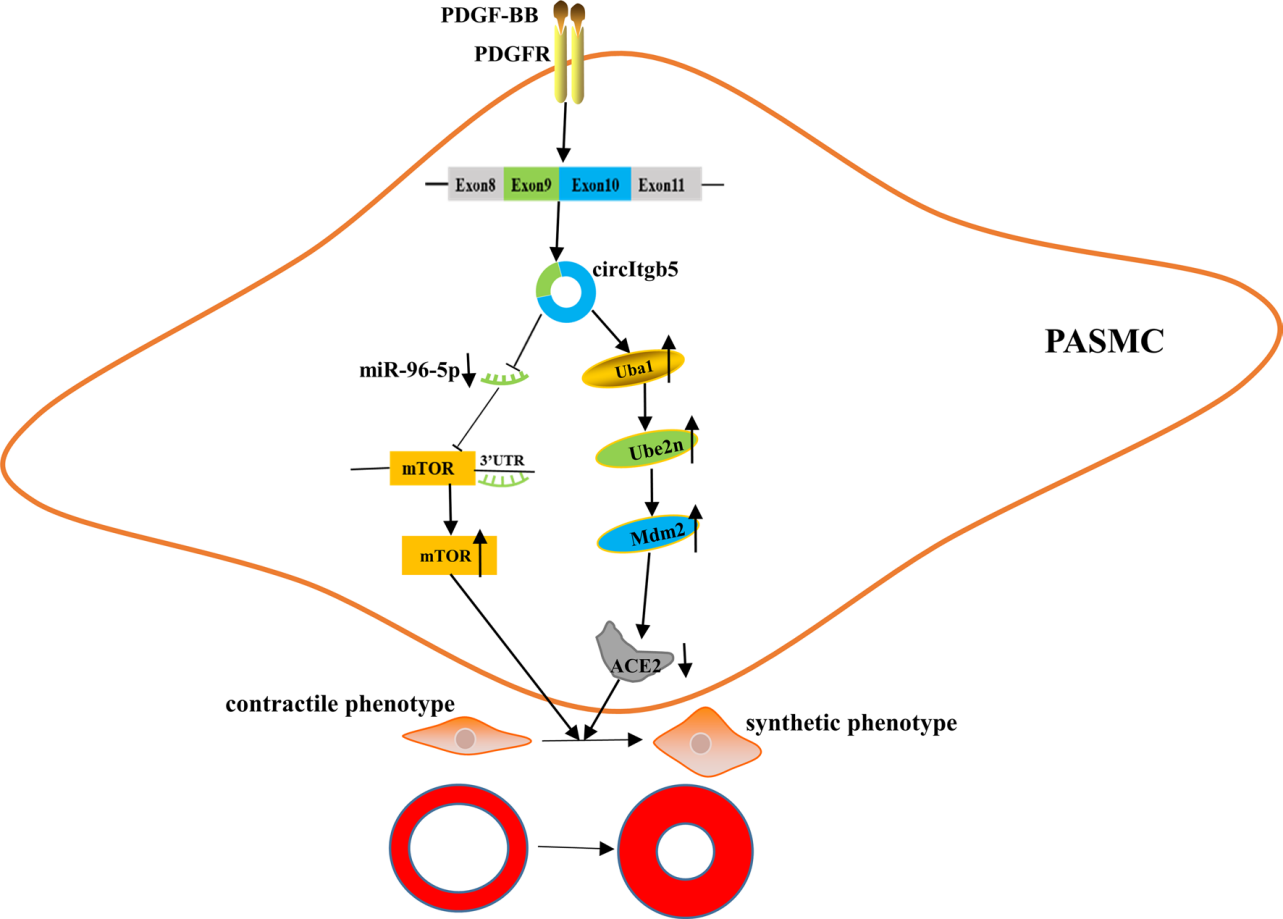
Integrated model depicting circItgb5 promoting pulmonary artery remodeling. The expression of circItgb5 was abnormally upregulated by PDGF-BB stimulation. Abnormally elevated circItgb5 levels can activate two pathways. On the one hand, circItgb5 sponges miR-96-5p to inhibit its activity. Decreased expression of miR-96-5p upregulates mTOR level, which promotes synthetic phenotype of PASMCs. On the other hand, circItgb5 enhances Uba1 protein expression, following Ube2n and Mdm2 activation. Mdm2 decreased ACE2 expression. Taken together, circItgb5 promotes synthetic phenotype of PASMCs, eventually resulting in pulmonary artery remodeling

In this study, we demonstrated that circItgb5-a novel circRNA found in PASMCs, was involved in the pathogenesis of PAH. Our findings were based on several key observations. First, circItgb5 was identified from high-throughput sequencing of circRNAs and was significantly upregulated by PDGF-BB stimulation. Second, in vivo silencing of circItgb5 attenuated MCT-induced pulmonary vascular remodeling and right ventricular hypertrophy. Third, the knockdown of circItgb5 inhibited synthetic phenotype of PASMCs. Finally, circItgb5 was found to function as a miRNA sponge to regulate the miR-96-5p/mTOR pathway and acted as a protein decoy to interact with Uba1, thereby activating the Ube2n/Mdm2/ACE2 pathway. Overall, these results demonstrated that circItgb5 regulated synthetic phenotype of PASMCs via interacting with miR-96-5p and Uba1 (Fig. 9).

PVR is a major pathological feature observed during the development of PAH. It has been established that uncontrolled growth of PASMCs is essential to PVR [18]. Current findings suggest that many circRNAs are dysregulated in PH and may be involved in regulating the proliferation of PASMCs, leading to PVR [6]. However, most studies on circRNAs have mainly focused on hypoxia-induced animals and cell models. For example, the expression of circ_calm4 was upregulated in hypoxia-induced PASMCs, and the circ_calm4/miR-337-3p/Myo10 pathway enhanced PASMCs proliferation [19]. Moreover, the circATP2B4/miR-223/ATR axis has been reported to regulate the proliferation, migration, and apoptosis of PASMCs under hypoxia [20]. Although some studies have evaluated circRNAs in PAH, few have demonstrated the role of PDGF-BB-induced circRNAs in PAH. PDGF-BB is a potent mitogen that contributes to PVR [21]. PDGF-BB can accelerate the alteration of PASMCs from a quiescent state toward a proliferative and synthetic phenotype, which is a key causative factor in the occurrence and progression of PAH [22]. The PDGF receptor antagonist, imatinib, could alleviate PVR in some patients, but severe adverse events still were observed [23]. Therefore, it is vital to explore the potential therapy targets and molecular mechanisms. In this study, we first found that circItgb5 was upregulated via PDGF-BB stimulation. We further demonstrated that circItgb5 was expressed mainly in the cytoplasm. Thus, we suggest that circItgb5 may regulate corresponding pathophysiological processes through complex mechanisms occurring in the cytoplasm.

CircRNAs, as endogenous RNAs, can act as miRNAs sponges to regulate the expression of target mRNAs. MiRNAs such as miR-221, miR-15b, and miR-96 have been reported to play a crucial role in the occurrence and development of PH by regulating PASMCs phenotypic switch, which is expected to be a potential target for the treatment of PH [5]. MiR-96 was previously reported to suppress the expression of Trb3. Suppression of the miR-96-Trb3 axis induced the conversion of PASMCs into a contractile phenotype [24]. Given that research on miR-96 regulating the pathological process of PAH is limited, exploring the potential targets of miR-96-5p is essential. In our study, miR-96-5p was sponged by circItgb5 and mTOR was a new target of miR-96-5p. Overall, we provided a novel molecular insight into miR-96-5p impacting PASMCs proliferation by suppressing mTOR expression. Consistently, it has been reported that activation of mTOR signaling ultimately results in abnormal proliferation of PASMCs [25]. We provide hitherto undocumented evidence that circItgb5 competitively sponges miR-96-5p with mTOR mRNA.

Mechanistically, emerging evidence has revealed that a group of circRNAs can serve as protein decoys, scaffolds and recruiters [7]. However, the understanding of circRNA-protein interactions in the context of PAH is limited, with only a few studies investigating this aspect [26]. A recent study revealed that circ_calm4 promotes hypoxia-induced PASMCs autophagy by forming a complex with Purb to enhance its expression in the nucleus [27]. Another study revealed that circ-Grm1 could promote the proliferation and migration of PASMCs by suppressing Grm1 expression through competitively binding to FUS [28]. Apart from these two studies, no other reports have investigated circRNA-protein interactions in the context of PAH. Thus, our study is the first to demonstrate that circItgb5 acted as a sponge for miR-96-5p and influenced the Uba1 protein level. Uba1 is the major E1 enzyme that initiates ubiquitylation [29]. The ubiquitin–proteasome system (UPS) is a post-translational mechanism that regulates cellular processes through modulation of protein stability [30, 31]. UPS has also been reported to regulate PAH-related proteins and cell proliferation [32]. For example, 15-LO2 is highly ubiquitinated by hypoxia stimulation, increasing the proliferation of PAECs [33]. In a previous study, many proteins were identified to have altered protein ubiquitination levels in response to hypoxia. These proteins were found to play a regulatory role in the proliferation of PASMCs in pulmonary hypertension [32]. Although it has been established that Uba1 is necessary for the initiation of ubiquitylation and is required for nearly all cellular ubiquitin signaling [29], the molecular mechanism underlying the posttranscriptional modification of Uba1 remains elusive. Our study indicated that circItgb5 promoted synthetic phenotype of PASMCs by interacting with Uba1. Further, we found that Uba1 interacted with Ube2n and Mdm2. Ube2n catalytic function is essential for transferring ubiquitin from a requisite E3 ubiquitin ligase to its substrates through the formation of ubiquitin chains [34]. A previous study confirmed that MDM2 expression was increased in the lung tissues of patients with PAH and animals with experimental PAH. Moreover, it has been shown that MDM2-mediated ubiquitination of ACE2 at K788 could lead to ACE2 degradation in PAECs. [17]. Similarly, our study observed an interaction between Mdm2 and ACE2 in PASMCs. Importantly, this study is the first to demonstrate that circItgb5 regulates the level of ACE2 through the E1 (Uba1)-E2 (Ube2n)-E3 (Mdm2) enzyme thioester cascade. However, we did not investigate the ubiquitination of ACE2 in this study, which is an area worth further exploration.

## Conclusions

In conclusion, this study provides compelling evidence that circItgb5 plays a significant role in driving the phenotypic switch of PASMCs both in vivo and in vitro. CircItgb5 achieves its function by acting as a sponge for miR-96-5p and interacting with Uba1. Additionally, we identified mTOR as a novel target of miR-96-5p in this context. Moreover, circItgb5 is also involved in regulating ACE2 expression through interacting with Uba1, thus influencing the Ube2n-Mdm2 enzyme thioester cascade. This study offers new insights into the molecular mechanism underlying the regulation of PDGF-BB-induced phenotypic switch in PASMCs. The role of circRNAs as protein decoys, scaffolds, or recruiters emerges as a crucial aspect for further investigations in the field of PAH. Research on circRNAs may provide a new potential target for the therapy of this patient population. Indeed, future studies are essential to investigate the relationship between circItgb5 level and the outcome of PAH patients and validate our findings at the clinical level.

## Supplementary Information

**Additional file 1.** Primer sequences.

**Additional file 2: Figure S1.** CircItgb5 expression in pulmonary artery endothelial cellsand lungs.Presence of circItgb5 was detected by RT-PCR in PASMCs and PAECs. GAPDH was used as a positive control. C, control; B, PDGF-BB.CircItgb5 expression in control and MCT-induced lungs. n = 6. *0.01 ≤ *P* ≤ 0.05 versus control. All data are presented as means ± SD. **Figure S2.** Knockdown of circItgb5 in control rats has no significantly effect on RVSP or morphology change of pulmonary arteries.RVSP in control and Lsh-circItgb5-treated groups. n = 6. Scale bar = 1 s.HE staining of small pulmonary arteries in control and Lsh-circItgb5-treated groups.Wall thicknessand muscularization of small pulmonary arteries. n = 6. NM, nonmuscularized; PM, partially muscularized; FM, fully muscularized.

**Additional file 3: Figure S3.** Original gel of Fig. 1D. **Figure S4.** Original blot images of Fig. 3C. **Figure S5.** Original blot images of Fig. 4B. **Figure S6.** Original gel of Fig. 5A. **Figure S7.** Original blot images of Fig. 6E. **Figure S8.** Original blot images of Fig. 6F. **Figure S9.** Original gel of Fig. 7A. **Figure S10.** Original gel of Fig. 7B. **FigureS11.** Original blot images of Fig. 7D. **Figure S12.** Original blot images of Fig. 7F. **Figure S13.** Original blot images of Fig. 8A. **Figure S14.** Original blot images of Fig. 8C. **Figure S15.** Original gel of Figure S1A.

## Data Availability

The raw high-throughput circRNAs sequencing data have been deposited in the Genome Sequence Archive (GSA: CRA010798) publicly accessible at https://ngdc.cncb.ac.cn/gsa. The datasets supporting the conclusions of this article are included within the article and its additional files.

## Abbreviations

CircItgb5: Circular integrin subunit beta 5
PH: Pulmonary hypertension
PAH: Pulmonary arterial hypertension
PVR: Pulmonary vascular remodeling
ECM: Extracellular matrix
PASMCs: Pulmonary arterial smooth muscle cells
PDGF-BB: Platelet derived growth factors beta polypeptide b
CircRNAs: Circular RNAs
miRNAs: MicroRNAs
MCT: Monocrotaline
SD: Sprague–Dawley
RT-qPCR: Reverse transcription-quantitative polymerase chain reaction
gDNA: Genomic DNA
cDNA: Complementary DNA
Lsh-NC: Lentiviral small hairpin RNA negative control
iCon: Inhibitor control
RIP: RNA immunoprecipitation
FISH: Fluorescence in situ hybridization
IHC: Immunohistochemistry
HE: Hematoxylin and eosin
RVSP: Right ventricular systolic pressure
RVHI: Right ventricular hypertrophy index
RV: Right ventricular
LV: Left ventricular
S: Ventricular septum
PPI: Protein–protein interaction
NM: Nonmuscularized
PM: Partially muscularized
FM: Fully muscularized
AGO2: Argonaute RNA-induced silencing complex catalytic component 2
PA: Pulmonary arteries
PAECs: Pulmonary artery endothelial cells
UPS: Ubiquitin–proteasome system
WT: Wild-type
MUT: Mutant
