# Supplementary material for: CircItgb5 promotes synthetic phenotype of pulmonary artery smooth muscle cells via interacting with miR-96-5p and Uba1 in monocrotaline-induced pulmonary arterial hypertension
Source: Respir Res. 2023 Jun 21;24:165. doi: 10.1186/s12931-023-02480-9 (PMC10283203; doi:10.1186/s12931-023-02480-9)
Supplement: Supplementary file 1 — Additional file 1. Primer sequences. [file 12931_2023_2480_MOESM1_ESM.docx]

**Table S1. PCR primers of circRNAs**

| circRNAs |  | Primer Sequence (5’ to 3’) |
| --- | --- | --- |
| circItgb5 | Forward | GCTCAGAGCATCCGGGCTAA |
|  | Reverse | TGTCCCCGATCTTCAGACCC |
| circReb1 | Forward | TCGCATTGGCAGAAGGAACC |
|  | Reverse | AGTTGGAACAACCTGACGGA |
| circVangl1 | Forward | AGAAGCGGAGAGCGAGCATC |
|  | Reverse | ATGCTGTTAGGAGGTTCGGGT |
| circCand2 | Forward | TGGGCAAGGTGAAGGAGTAC |
|  | Reverse | CTTACGCTCGCTGTCCTCAT |
| circMvp | Forward | AGGAAGTGGAAGTCGTGGAG |
|  | Reverse | AAGATGCAGTGCTGTGTTGG |
| circSchip1 | Forward | AAGAGAGCCATCCACCAGAG |
|  | Reverse | GGCCATCGTCAAAGAAGCTT |
| circGucyla2 | Forward | TATGTGCCCAGTGTACTCCC |
|  | Reverse | GTCCTCGGCCTATGAGTTCA |
| circMmd | Forward | GGTGACATCAATGTTCCTCATCGT |
|  | Reverse | ACAAAGGCCCATCCCGTAGA |
| circLpar1 | Forward | GGCTATGTTCGCCAGAGGAC |
|  | Reverse | GCTGTGAACCAGCACAATGAC |
| circExoc2 | Forward | TGATATGCGGACTGACAGGA |
|  | Reverse | CCAGTACCCAGATTCTCCCC |
| circWdr33 | Forward | GCTCCCCGACAGTTGTTCTA |
|  | Reverse | ATTGCCCGCATATCTCTCTG |
| circSyndig1 | Forward | GTGATGCTGGCAAAAGGAAT |
|  | Reverse | TTGGGACGATAGTGGGAGTC |
| circDiaph3 | Forward | CTTGAAGCAGATGGGAAGGC |
|  | Reverse | CGTGTCGACAGCATAGTAGC |
| circTimmdc1 | Forward | GGAGTTGGAGAACCGCAGTC |
|  | Reverse | CCGCTGTTCACTCCAGCAAT |
| circPbx3 | Forward | CTCCATCCAAATGCAGCTCA |
|  | Reverse | ATTTCTTGGCCAGCTCCTCT |
| circAuh | Forward | GTCTCCAAGATCCGAGCAGT |
|  | Reverse | ATCGCCAACTTCGTTTCCAC |
| circCacna1d | Forward | AACTCGTCTAACAGCCGACC |
|  | Reverse | ATCTTCAGGGAACGGGATGT |
| circRasa2 | Forward | TATCGCACCCAGGTTGTTGA |
|  | Reverse | AGGCTGCAATGAAAACCAAGT |
| circStag1 | Forward | TCTGATGCTGGCAGCGAACTT |
|  | Reverse | ACCTCAAAGCCACACTGGAGG |
| circLtp1 | Forward | TCAAGTTGTGTGCTGTGGCA |
|  | Reverse | TCTTGGGGCATTTCTGGCATT |
| *Continued……* |  |  |
| *Continued……* | | |
| circRNAs |  | Primer Sequence (5’ to 3’) |
| circSpidr | Forward | GCCTCCTCCTGTCTTCTGTT |
|  | Reverse | AAGCTGCAACGTGTACCATG |

**Table S2. PCR primers of Genes**

| Genes |  | Primer Sequence (5’ to 3’) |
| --- | --- | --- |
| Itgb5 | Forward | AACATACGTCTGTGGGCTGT |
|  | Reverse | CCGTAGATCCTCCCGAACTC |
| COL1A1 | Forward | AAGGCAATGCTGAATCGTCC |
|  | Reverse | TGTGTGGGGATTTGTTGCAG |
| syndecan-1 | Forward | ATGTGCCTCCTGAAGACCAA |
|  | Reverse | TTCTGGAGCTGTGGGTGTAG |
| SM22α | Forward | AGATCATGCTGTGGTGTCCA |
|  | Reverse | AAGCAGGTTGGAGGAGAGAC |
| α−SMA | Forward | CATCATGCGTCTGGACTTGG |
|  | Reverse | CCAGGGAAGAAGAGGAAGCA |
| GAPDH | Forward | GAGACAGCCGCATCTTCTTG |
|  | Reverse | TGACTGTGCCGTTGAACTTG |
| Smad1 | Forward | TGGGCTGCTCTCCAATGTTA |
|  | Reverse | AGATTTTCAAGCTGCACCCG |
| Mmp9 | Forward | AGCGAGACACTAAAGGCCAT |
|  | Reverse | CCAGCCTCTCGGAACTTGTA |
| Id1 | Forward | CTCTGAGTCTGAAGTCGCGA |
|  | Reverse | GTAGTGTCTTTCCCCGGGTT |
| Stat3 | Forward | TGATACAGGGGCATGGCTAG |
|  | Reverse | GGGAAGGGAGAGCAATGACT |
| Tgfb1 | Forward | TCGCTTTGTACAACAGCACC |
|  | Reverse | ACTGCTTCCCGAATGTCTGA |
| mTOR | Forward | AGGAAGGACGTTTGCTCAGA |
|  | Reverse | ATTCACAACCTGCGCTAGTG |

**Table S3. PCR primer sequence of miRNAs and siRNAs sequence**

| Primers |  | Primer Sequence (5’ to 3’) |
| --- | --- | --- |
| rno-miR-96-5p |  | TTTGGCACTAGCACATTTTTGCT |
| rno-miR-217-5p |  | TACTGCATCAGGAACTGACTGG |
| rno-let-7g-3p |  | CTGTACAGGCCACTGCCTTGC |
| rno-miR-143-5p |  | GGTGCAGTGCTGCATCTCTGG |
| rno-miR-376a-5p |  | GGTAGATTCTCCTTCTATGAG |
| rno-miR-346 |  | TGTCTGCCTGAGTGCCTGCCTCT |
| rno-miR-196b-3p |  | TCGACAGCACGACACTGCCTTCA |
| rno-miR-455-3p |  | GCAGTCCACGGGCATATACACT |
| rno-miR-96-5p Inhibitor |  | AGCAAAAATGTGCTAGTGCCAAA |
| Inhibitor control |  | UUUGUACUACACAAAAGUACUG |
| 5sRNA |  | GTCTACGGCCATACCACCCTGAAC |
| universal r |  | GCTGTCAACGATACGCTACGTAACG |
| Anchor RT |  | GCTGTCAACGATACGCTACGTAACGGCATGACAGTG－ttttttttttttttttttttttttG |
| Mimic control | Forward | UUUGUACUACACAAAAGUACUG |
|  | Reverse | CAGUACUUUUGUGUAGUACAAA |
| rno-miR-96-5p mimic | Forward | UUUGGCACUAGCACAUUUUUGCU |
|  | Reverse | AGCAAAAATGTGCTAGTGCCAAA |
| Lsh-circItgb5 | Forward | CCUUCUGCGAGUGCGACAGCTT |
|  | Reverse | CUCACACUUCCUCUGACCAGTT |
| Lsh-NC | Forward | UUCUCCGAACGUGUCACGUTT |
|  | Reverse | ACGUGACACGUUCGGAGAATT |
