# Supplementary figures and images for: CircItgb5 promotes synthetic phenotype of pulmonary artery smooth muscle cells via interacting with miR-96-5p and Uba1 in monocrotaline-induced pulmonary arterial hypertension
Source: Respir Res. 2023 Jun 21;24:165. doi: 10.1186/s12931-023-02480-9 (PMC10283203; doi:10.1186/s12931-023-02480-9)

Supplementary Figure 1

A

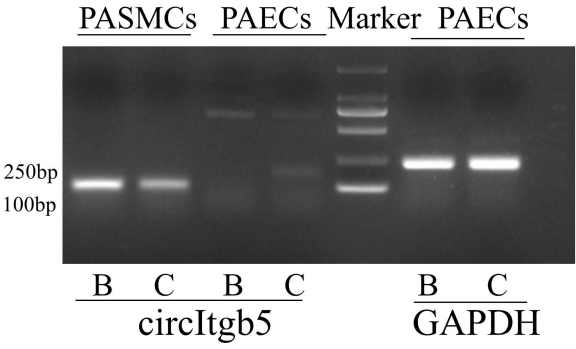

B

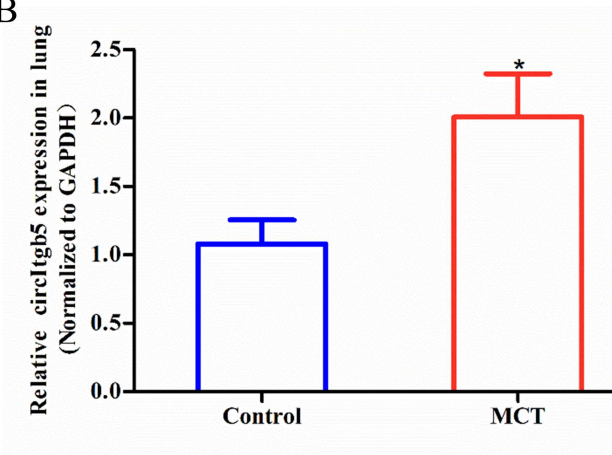

Supplementary Figure 2

A

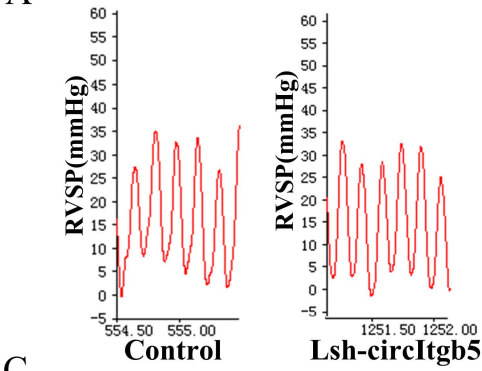

B

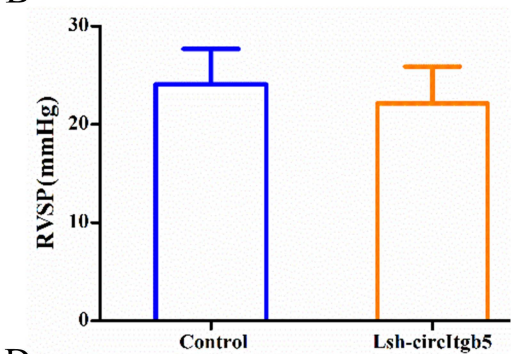

C

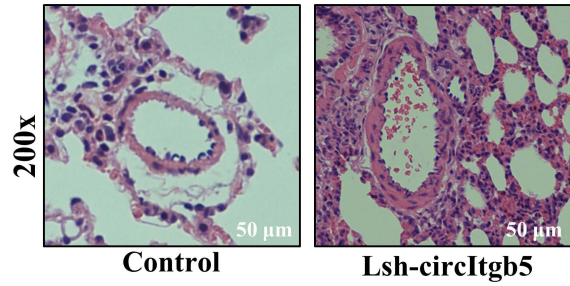

D

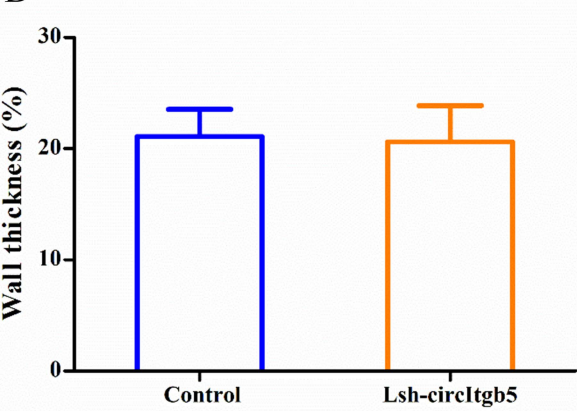

E

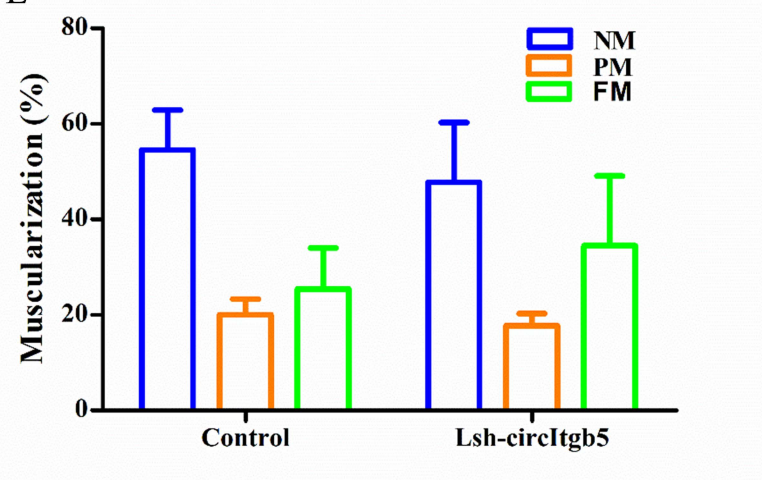

Supplement: Supplementary file 2 — Additional file 2: Figure S1. CircItgb5 expression in pulmonary artery endothelial cellsand lungs.Presence of circItgb5 was detected by RT-PCR in PASMCs and PAECs. GAPDH was used as a positive control. C, control; B, PDGF-BB.CircItgb5 expression in control and MCT-induced lungs. n = 6. *0.01 ≤ P ≤ 0.05 versus control. All data are presented as means ± SD. Figure S2. Knockdown of circItgb5 in control rats has no significantly effect on RVSP or morphology change of pulmonary arteries.RVSP in control and Lsh-circItgb5-treated groups. n = 6. Scale bar = 1 s.HE staining of small pulmonary arteries in control and Lsh-circItgb5-treated groups.Wall thicknessand muscularization of small pulmonary arteries. n = 6. NM, nonmuscularized; PM, partially muscularized; FM, fully muscularized. [file 12931_2023_2480_MOESM2_ESM.pdf]
