# Supplementary material for: CircItgb5 promotes synthetic phenotype of pulmonary artery smooth muscle cells via interacting with miR-96-5p and Uba1 in monocrotaline-induced pulmonary arterial hypertension
Source: Respir Res. 2023 Jun 21;24:165. doi: 10.1186/s12931-023-02480-9 (PMC10283203; doi:10.1186/s12931-023-02480-9)

Supplementary Figure 3

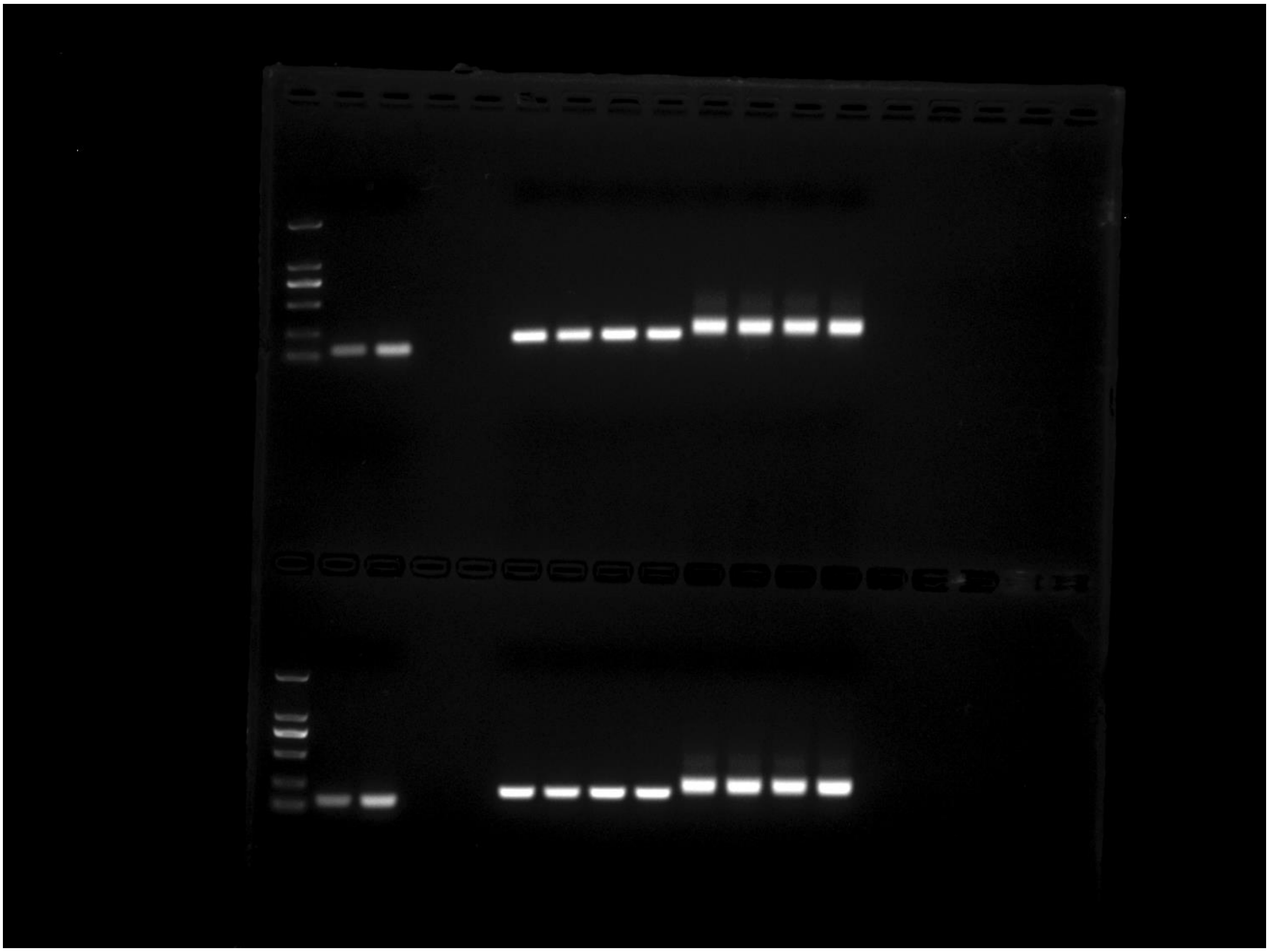

Supplementary Figure 4

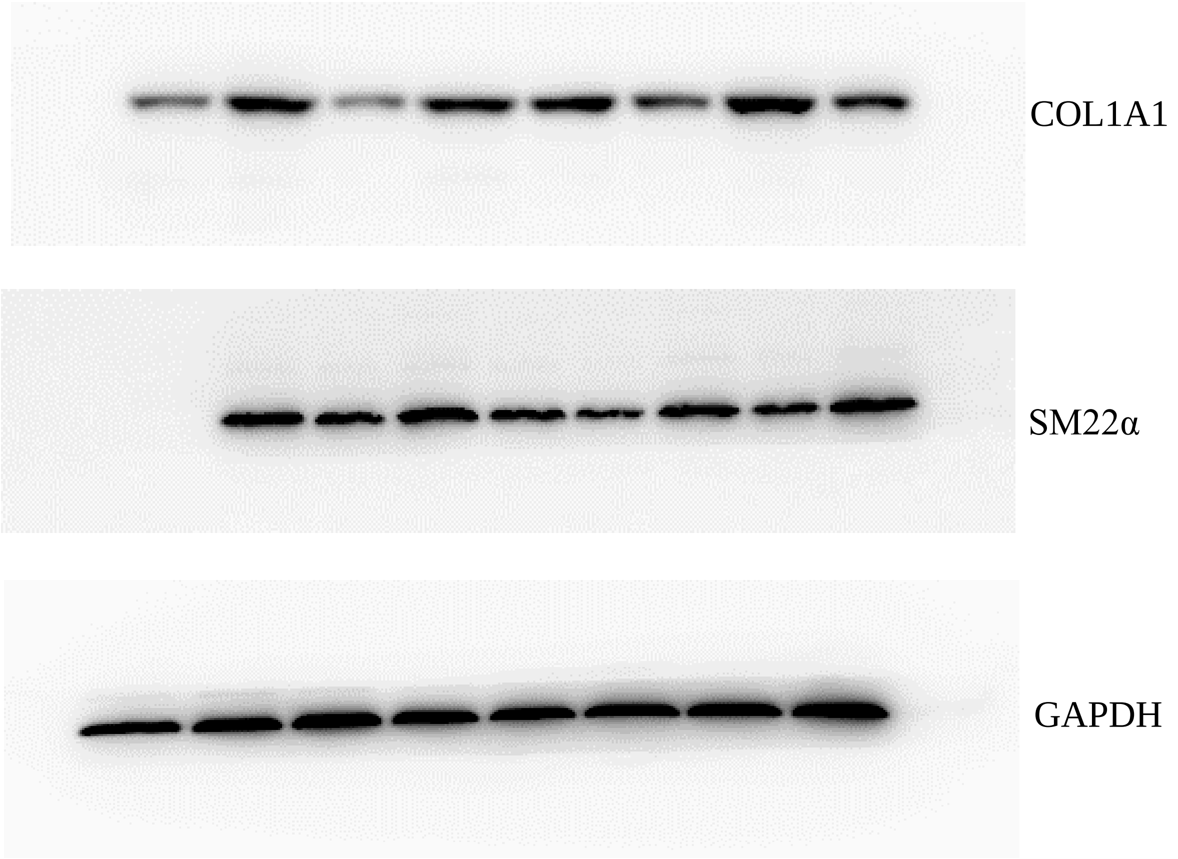

Supplementary Figure 5

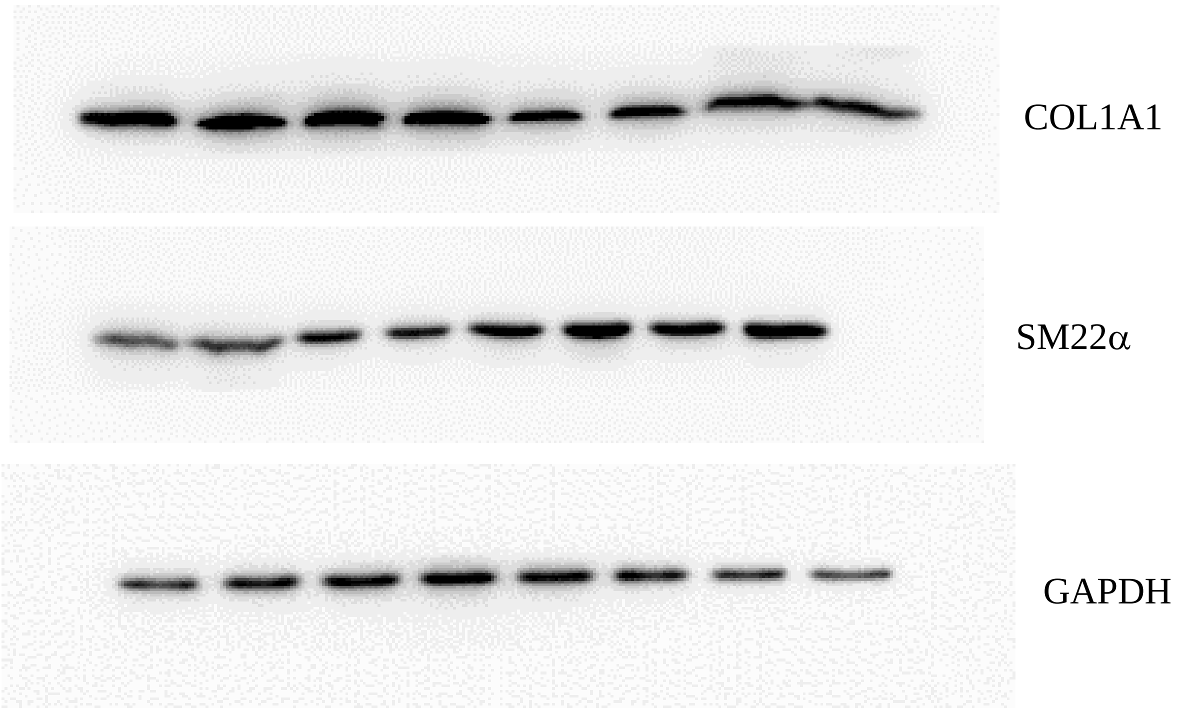

Supplementary Figure 6

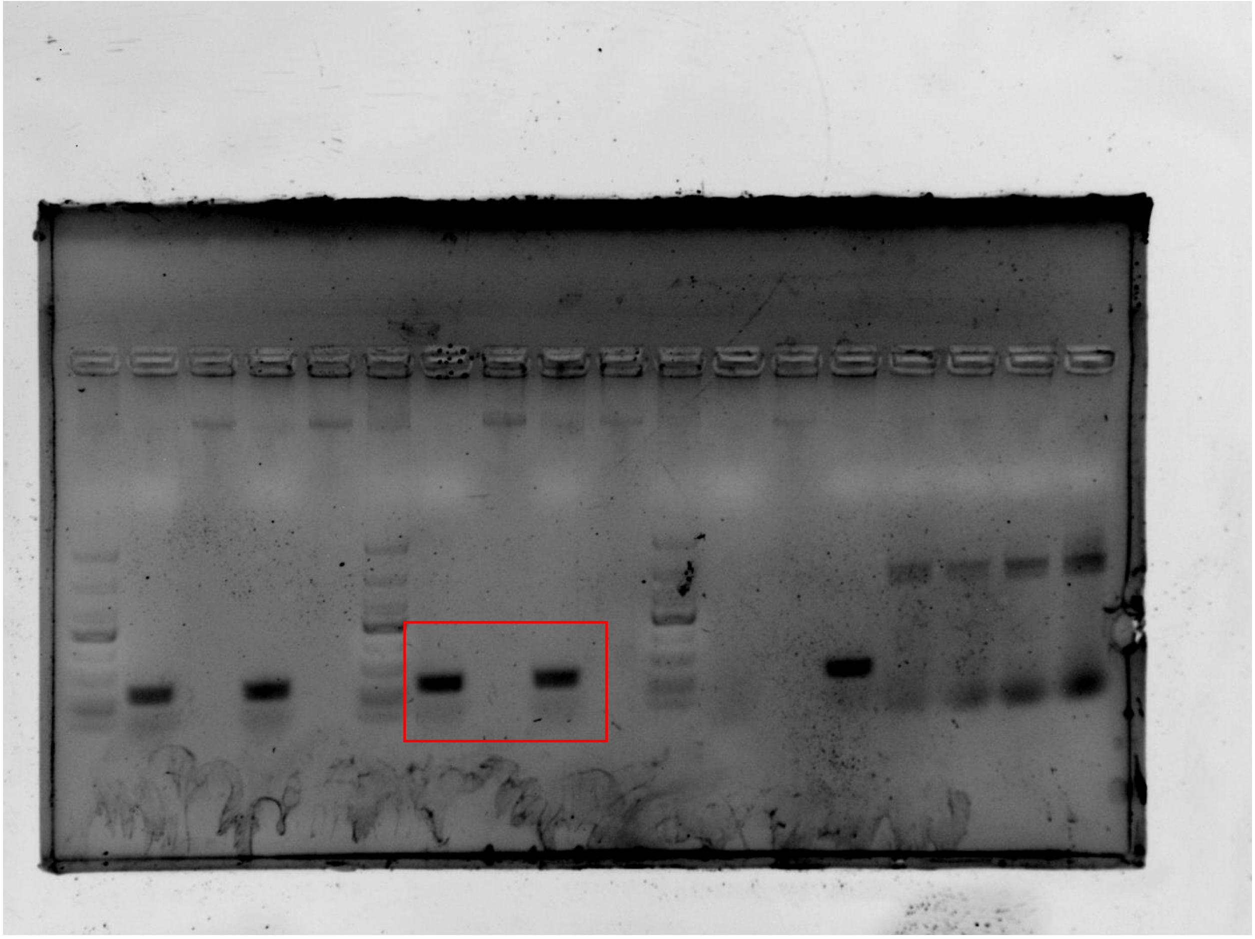

Supplementary Figure 7

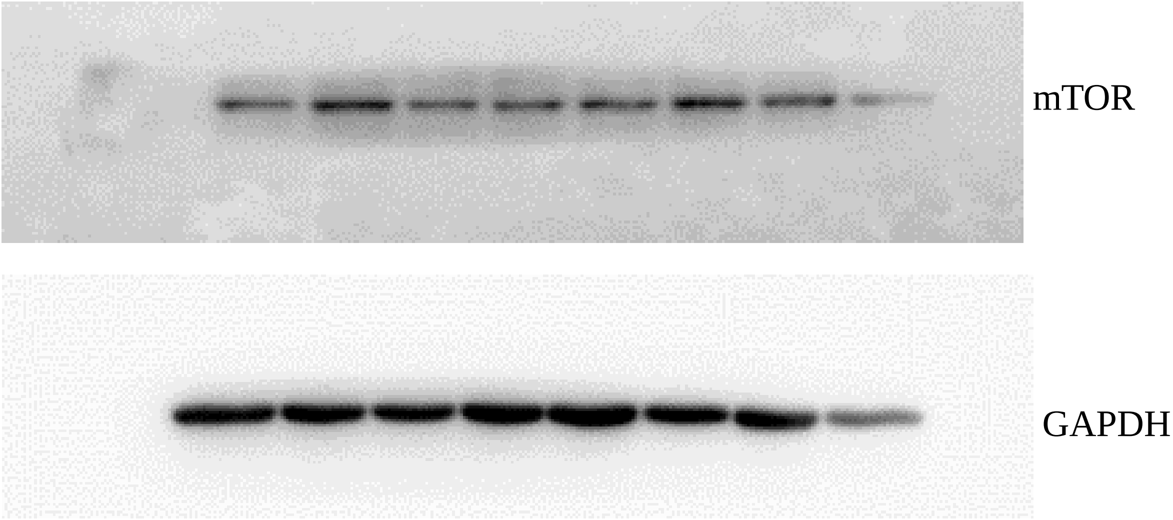

Supplementary Figure 8

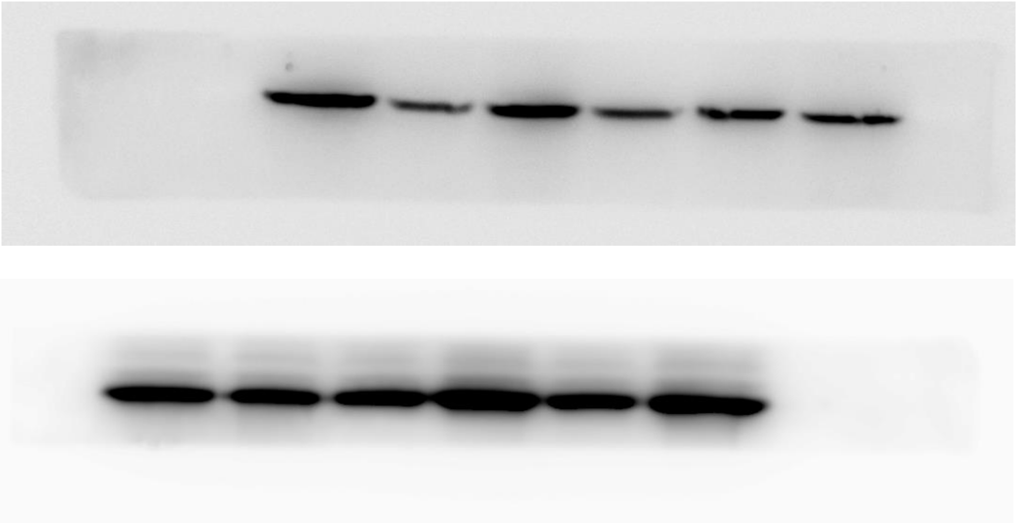

mTOR

GAPDH

Supplementary Figure 9

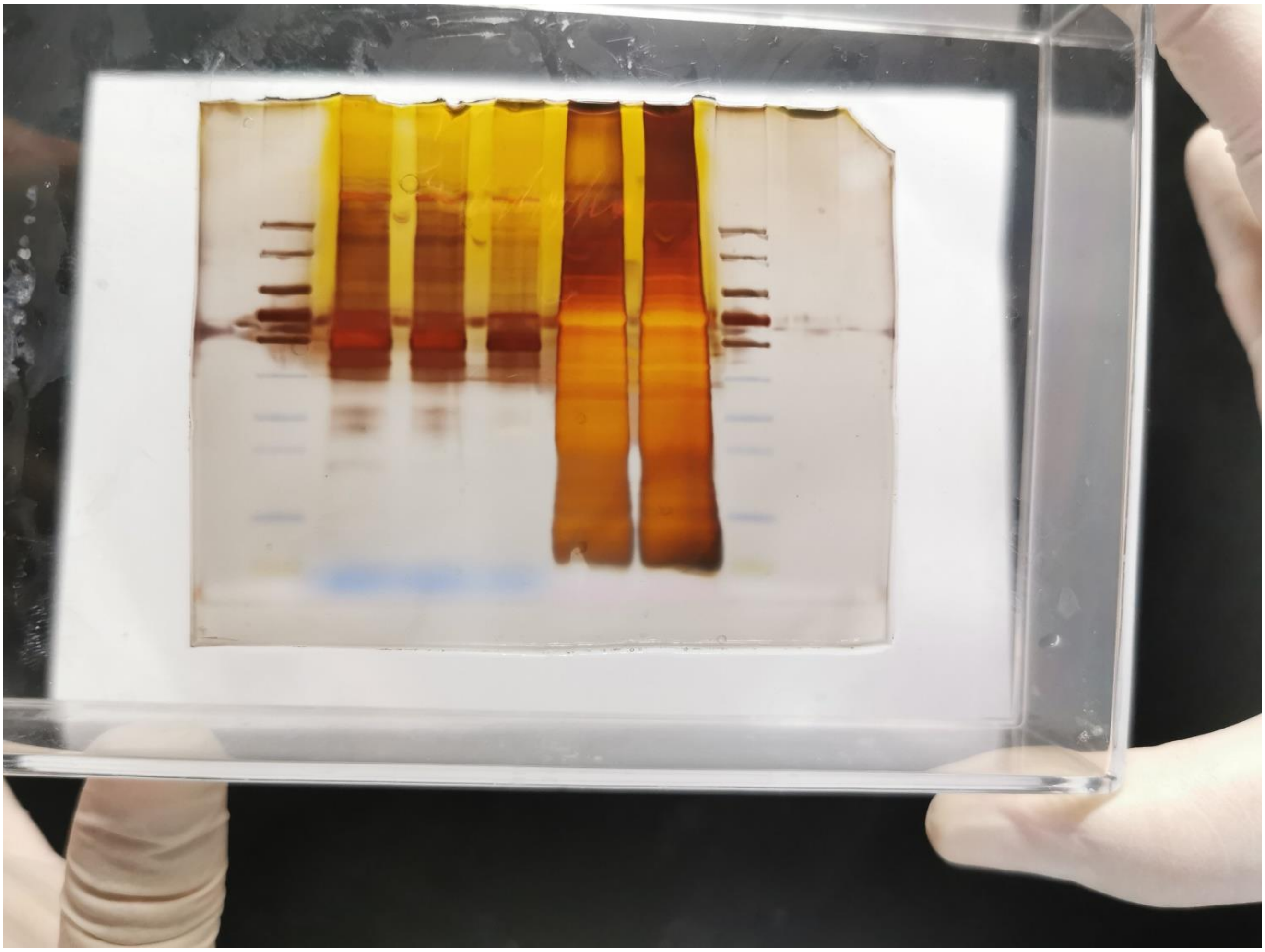

Supplementary Figure 10

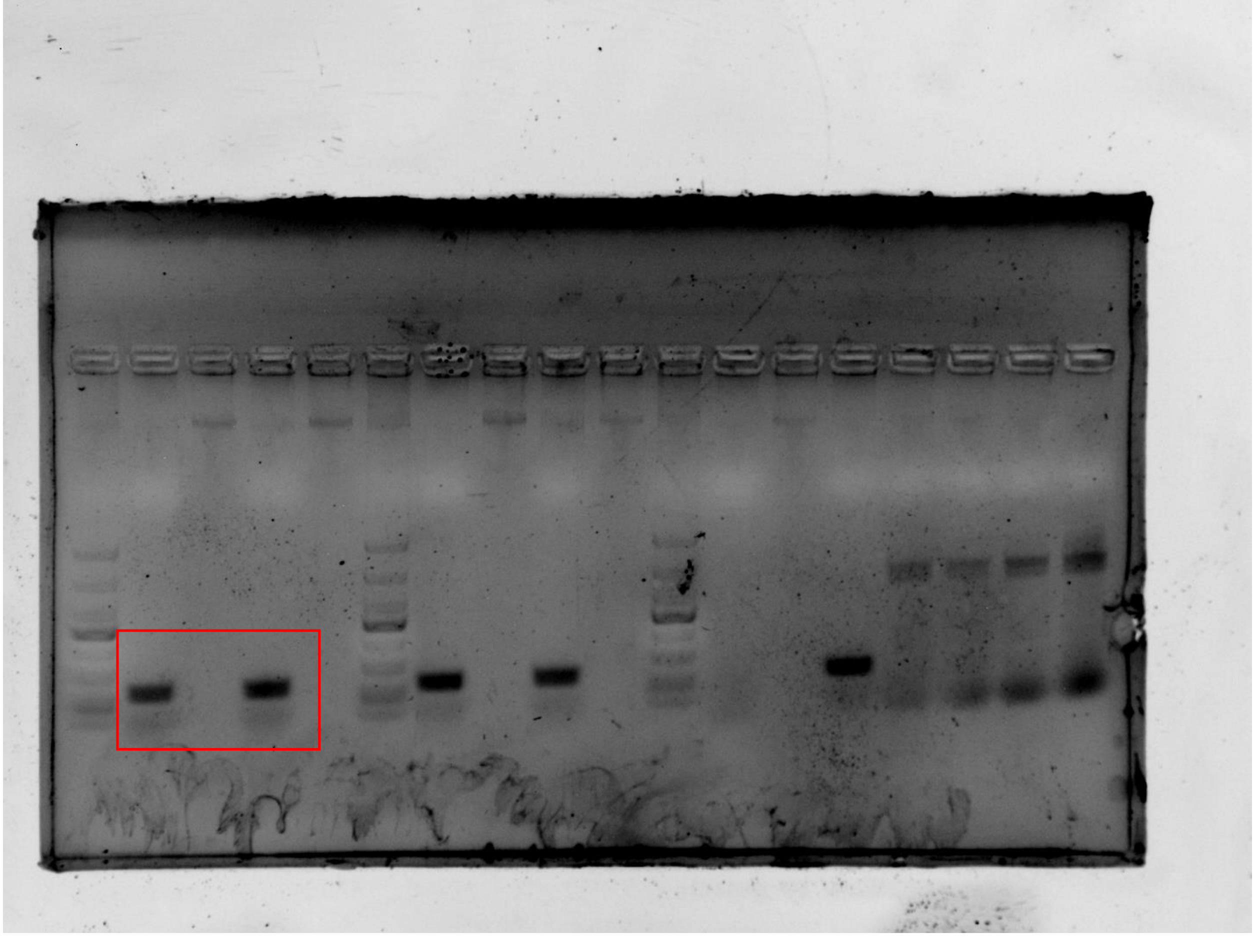

Supplementary Figure 11

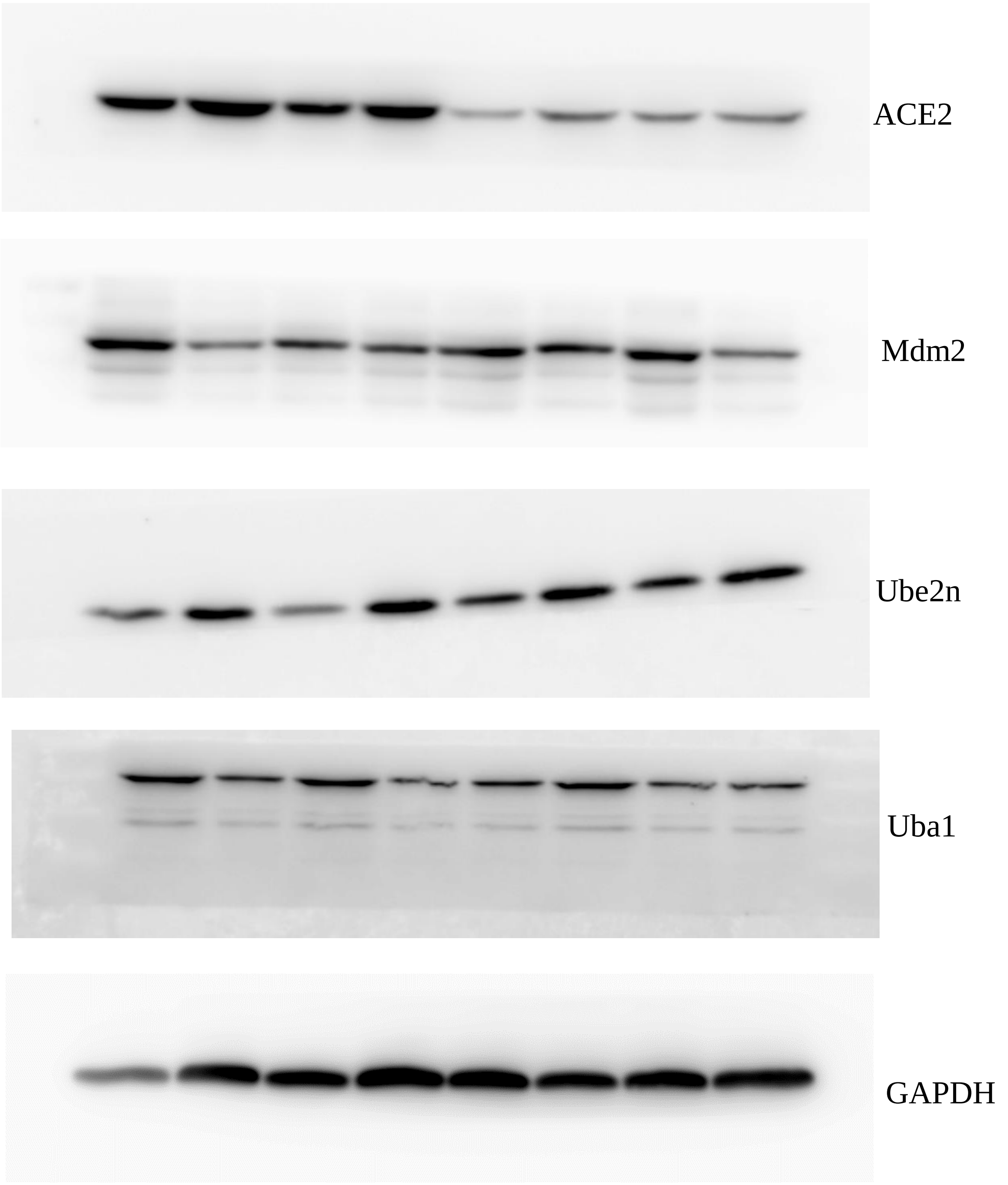

Supplementary Figure 12

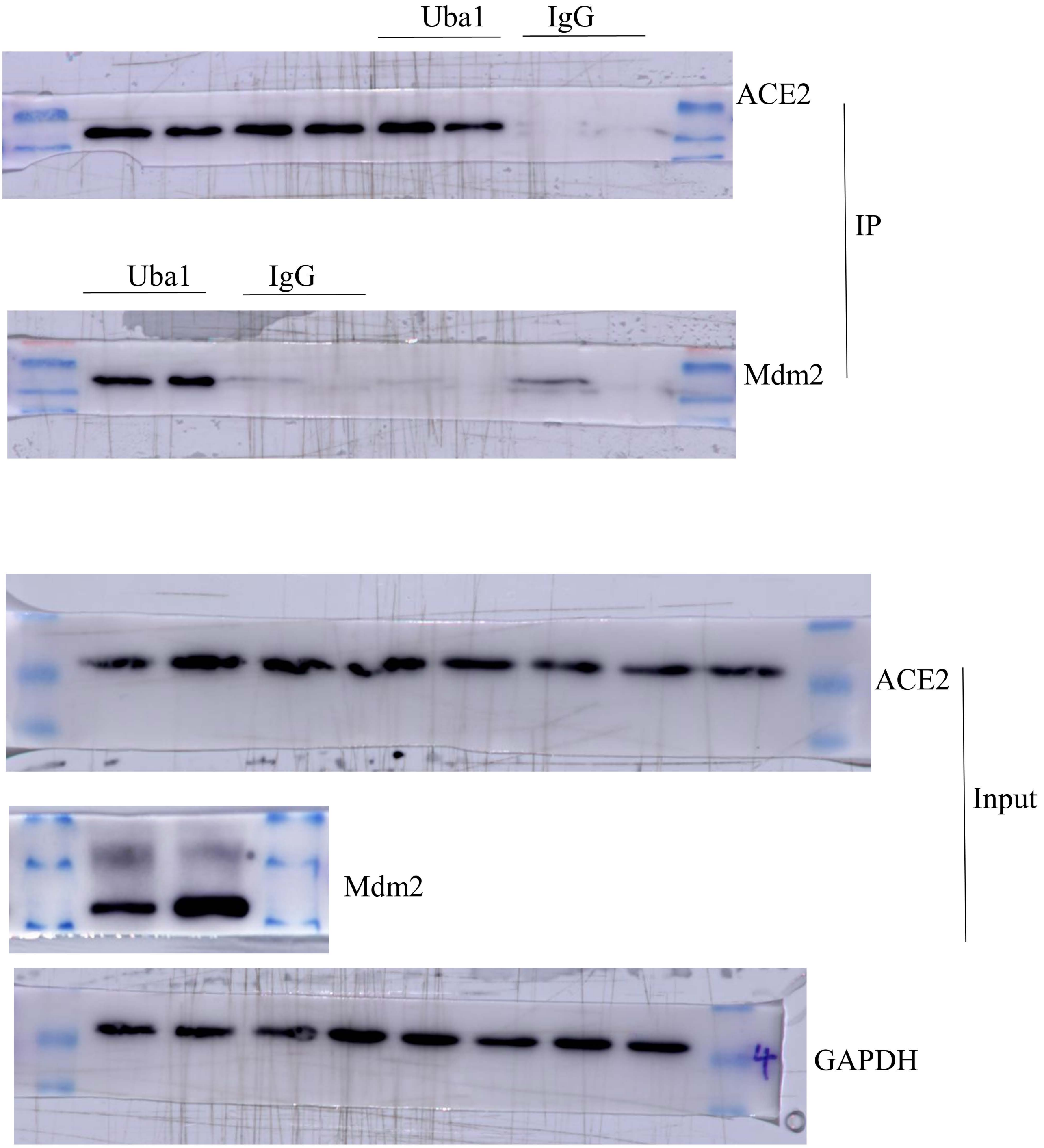

Supplementary Figure 13

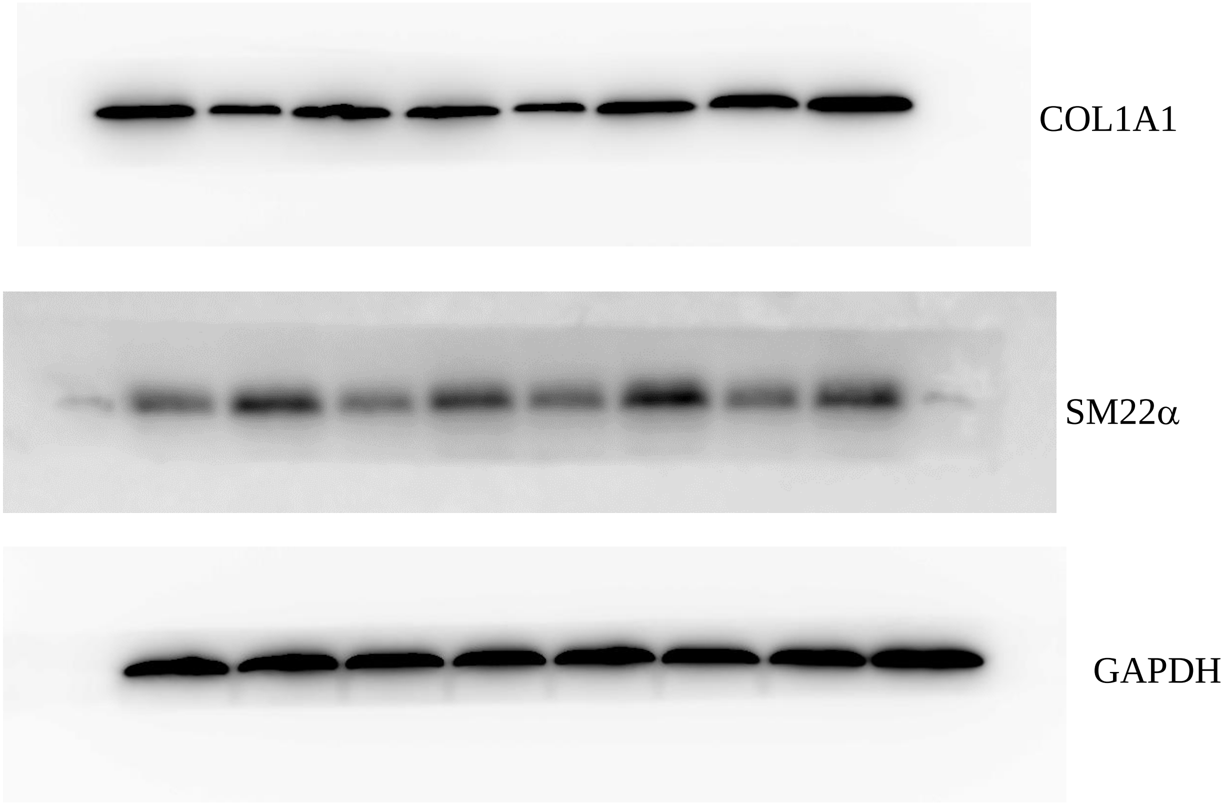

Supplementary Figure 14

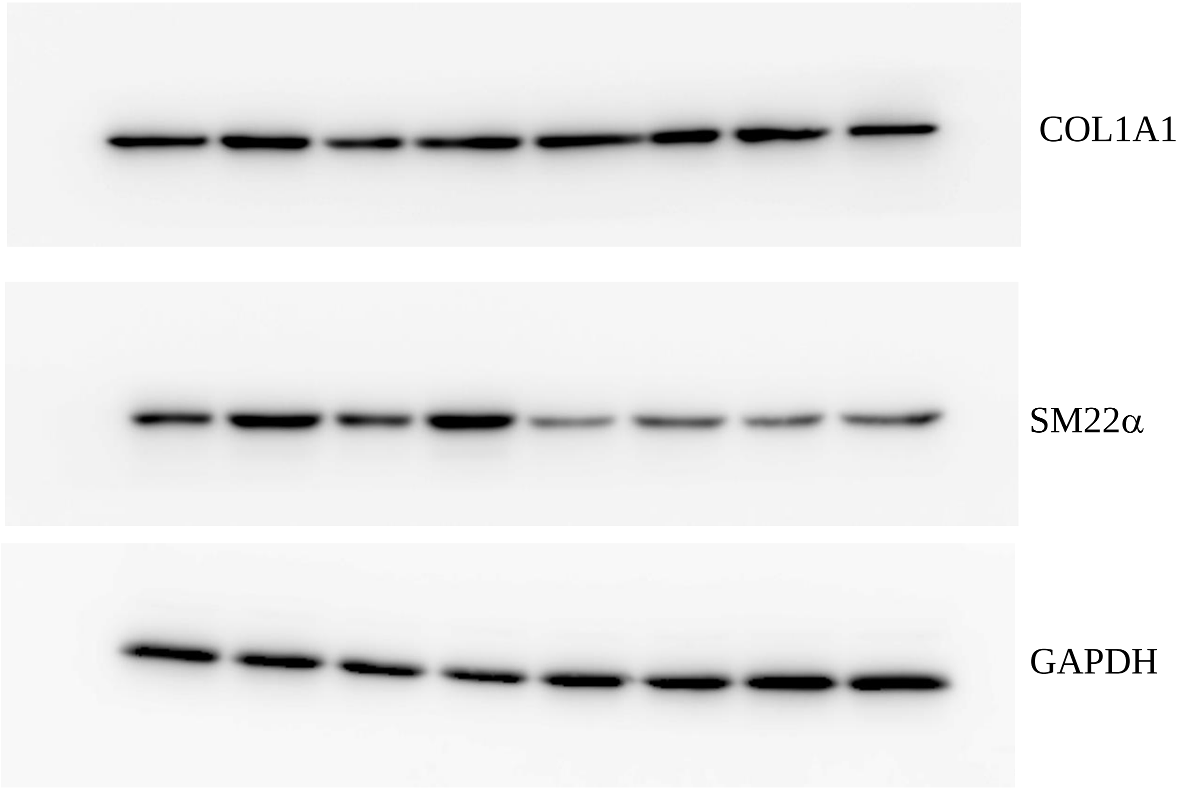

Supplementary Figure 15

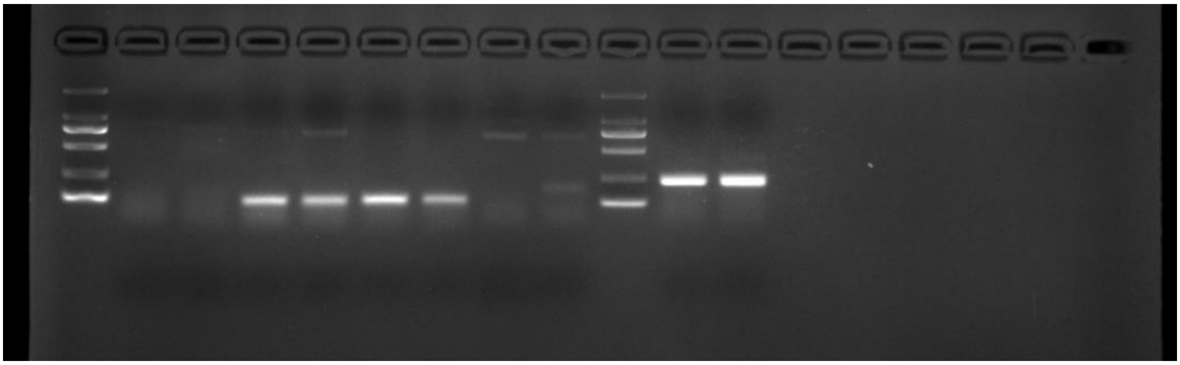

Supplement: Supplementary file 3 — Additional file 3: Figure S3. Original gel of Fig. 1D. Figure S4. Original blot images of Fig. 3C. Figure S5. Original blot images of Fig. 4B. Figure S6. Original gel of Fig. 5A. Figure S7. Original blot images of Fig. 6E. Figure S8. Original blot images of Fig. 6F. Figure S9. Original gel of Fig. 7A. Figure S10. Original gel of Fig. 7B. FigureS11. Original blot images of Fig. 7D. Figure S12. Original blot images of Fig. 7F. Figure S13. Original blot images of Fig. 8A. Figure S14. Original blot images of Fig. 8C. Figure S15. Original gel of Figure S1A. [file 12931_2023_2480_MOESM3_ESM.pdf]
